# Untangling the Extracellular Matrix of Idiopathic Epiretinal Membrane: A Path Winding among Structure, Interactomics and Translational Medicine

**DOI:** 10.3390/cells11162531

**Published:** 2022-08-15

**Authors:** Laura Bianchi, Annalisa Altera, Virginia Barone, Denise Bonente, Tommaso Bacci, Elena De Benedetto, Luca Bini, Gian Marco Tosi, Federico Galvagni, Eugenio Bertelli

**Affiliations:** 1 Department of Life Sciences, University of Siena, 53100 Siena, Italy; 2 Department of Molecular and Developmental Medicine, University of Siena, 53100 Siena, Italy; 3 Department of Medicine, Surgery and Neuroscience, University of Siena, 53100 Siena, Italy; 4 Department of Biotechnology, Chemistry and Pharmacy, University of Siena, 53100 Siena, Italy

**Keywords:** idiopathic epiretinal membranes, extracellular matrix, ITGB1, CTSB, EGFR, TGM2, LRP1, vitreolysis

## Abstract

Idiopathic epiretinal membranes (iERMs) are fibrocellular sheets of tissue that develop at the vitreoretinal interface. The iERMs consist of cells and an extracellular matrix (ECM) formed by a complex array of structural proteins and a large number of proteins that regulate cell–matrix interaction, matrix deposition and remodelling. Many components of the ECM tend to produce a layered pattern that can influence the tractional properties of the membranes. We applied a bioinformatics approach on a list of proteins previously identified with an MS-based proteomic analysis on samples of iERM to report the interactome of some key proteins. The performed pathway analysis highlights interactions occurring among ECM molecules, their cell receptors and intra- or extracellular proteins that may play a role in matrix biology in this special context. In particular, integrin β1, cathepsin B, epidermal growth factor receptor, protein-glutamine gamma-glutamyltransferase 2 and prolow-density lipoprotein receptor-related protein 1 are key hubs in the outlined protein–protein cross-talks. A section on the biomarkers that can be found in the vitreous humor of patients affected by iERM and that can modulate matrix deposition is also presented. Finally, translational medicine in iERM treatment has been summed up taking stock of the techniques that have been proposed for pharmacologic vitreolysis.

## 1. Introduction

The vitreoretinal interface is the border region where the vitreous meets the internal limiting membrane (ILM) of the retina [1]. In general, the vitreous does not adhere strongly to the ILM with the notable exception of a few important areas including the vitreous base, a perifoveal rim and the optic papilla [1,2]. The vitreoretinal interface is the site where a series of pathologies such as posterior vitreous detachment, macular holes (MH)s, vitreomacular traction and epiretinal membranes (ERM)s can develop [3]. ERMs, in particular, are formed by a fibrocellular layer of tissue that can seriously affect vision when developing and retracting in front of the macula [4]. ERMs are generated secondarily to systemic or local diseases (e.g., diabetes, retinal tears), or they can develop without any apparent reason. In the latter case, they are referred to as idiopathic ERMs (iERM)s. Although the cellular component of ERMs has been the object of several investigations and many molecular markers have been employed for cell identification, the source of ERM cells is a matter that still appears far from being solved [5,6,7,8,9,10,11,12]. Probably due to a process of transdifferentiation, ERM cells take on a myofibroblastic phenotype with the expression of α-smooth muscle actin (α-SMA) [5,6,7,8,9,10,11,12] and the ability to deposit new extracellular matrix (ECM) [11,12]. Conversely, less attention has been paid to the ECM of ERMs. As it is likely that distinct pathogenetic events may give rise to different tissue responses, we chose to focus this review on the ECM of iERMs, a subset of ERMs that is not secondary to any evident systemic or local disorder. Nevertheless, we will also refer to studies carried out on ERMs secondary to proliferative vitreoretinopathy (PVR), proliferative diabetic vitreoretinopathy (PDR) and to MHs mainly for comparative purposes. Our goal is to summarize what we currently know on the composition of the ECM of iERMs, trying to catch a glimpse on the emerging order of its molecular architecture. With a bioinformatic approach based on previously published results, we also outline the most likely and relevant molecular interactions that can lead to the deposition, organization and accumulation of the ECM which also influence iERM progression. Finally, we discuss current strategies of vitreolysis based on the degradation of the ECM in ERMs.

## 2. Materials and Methods

A Pubmed and Google search was carried out with the following primary keywords: “epiretinal membrane”, “proliferative vitreoretinopathy”, “preretinal macular fibrosis”, “macular pucker” or “vitreolysis”. Each primary keyword was cross-referenced with the following secondary keywords: “extracellular matrix”, “collagen”, “proteoglycan” or “glycosaminoglycan” (GAG). The reference lists of the relevant articles were further checked to extend the results of the electronic search.

By taking advantage of the most comprehensive and rigorous MS/MS-based proteomic approach we found in the literature for systematic identification of proteins from iERM ECM and cells [13], we performed a bioinformatics analysis to predict functional interactions occurring between iERM proteins. The 596 proteins identified in iERM specimens from 12 patients (8 males and 4 females, age range and mean: 55–82 and 70 years, respectively) by using LFQnLC-MS/MS [13] were filtered for the UniProtKB (http://www.uniprot.org/; 2021_03 release. Gene Ontology (GO) terms [recovered from the three ontologies, Biological Process, Molecular Function, and Cellular Component, by using the QuickGO browser (http://www.ebi.ac.uk/QuickGO/ accessed on 15 June 2021) containing the words “extracellular” and/or “matrix”. We also included proteins not annotated by these terms but rather by the “basement membrane” GO term. The list was finally restricted by excluding proteins annotated by “extracellular” and/or “matrix” in combination with “vesicle” and/or “exosome” terms. This bona fide selection method allowed us to process proteins taking physical part to the ECM composition or that may be involved in cellular and extracellular processes, signaling and dynamics concurring to the formation, remodeling and maturation of iERMs.

The selected 141 proteins, listed in Table S1, were functionally correlated by pathway analysis using the network building tool from the MetaCore (v. 21.2) integrated software suite for functional analysis of experimental data (Clarivate Analytics, London). MetaCore is based on a manually curated database of human protein–protein and protein–DNA interactions, signaling and metabolic pathways, in physiological and pathological conditions, from the scientific literature. Previously, we successfully applied it to predict biomarkers and/or related pathways for defining biochemical bases of the investigated systems, and we also proved the quality and reliability of the obtained results by performing targeted experiments [14,15,16,17].

Gene names of the selected unique proteins were imported into MetaCore and processed using the “direct interaction” and the “shortest path” algorithms. The former builds hypothetical networks by cross-linking experimental factors reported in the MetaCore in-house database as directly interacting. Nets were constructed limiting the protein process to individual proteins and excluding their involvement in multimeric complexes. The shortest path algorithm, in contrast, expanded protein interactions to other proteins not present in the processed experimental list but supported by the MetaCore database, which are needed to functionally correlate user up-loaded proteins that do not directly interact. The maximum number of steps cross-linking two experimental proteins via the software-added protein was set to 2, and protein complexes as well as canonical pathways were avoided. Generated pathway maps were then prioritized according to their statistical significance (*p* < 0.001), and the most significant direct interaction network (DIN) and shortest path network (SPN) were graphically visualized as nodes (proteins) and edges (the relationship between proteins). A wide literature search was then performed in PubMed/PubMed Central and Google Scholar for functional reviews of the outlined iERM protein cross-talks and pathways, thus to define their possible role in iERM occurrence and outcome. MetaCore usually refers to proteins using their gene names. Nevertheless, some proteins in the nets may appear with alternative names. To facilitate net protein recognition, Table S1 also supplies the MetaCore names of the 141 processed unique proteins as occurring in the DIN and SPN.

Finally, we performed an enrichment analysis on the selected 141 iERM proteins by using the freely available DAVID bioinformatics resource v. 6.8 (http://david.abcc.ncifcrf.gov accessed on 10 August 2021), merely to support MetaCore built nets. We hence limited our DAVID analysis to the three GOs and the dissertation to two significantly enriched Biological Process GO-terms: “proteolysis” and “response to reactive oxygen species”.

The mass spectrometry proteomics data were deposited in the Pro-teomeXchange Consortium via the PRIDE partner repository with the dataset identifier PXD0142 [13].

## 3. ECM Components of iERMs

The ECM of iERMs encompasses many collagens, basement membrane proteins, proteoglycans, GAGs, as well as some adhesive and matricellular proteins. In addition, collagens IX, XII, XIV, XVIII and XXII have been found by proteomic profiling [13].

### 3.1. Collagens

The major component of the iERM ECM is certainly represented by collagen. So far, only collagens I to VII have been detected in iERMs by immunohistochemistry. In addition, collagens IX, XII, XIV, XVIII and XXII have been found by proteomic profiling [13].

#### 3.1.1. Collagen I

Collagen I has been repetitively reported in iERMs [12,18,19,20,21], and it is certainly shared with other types of ERMs [22,23,24]. As collagen I is a hallmark of all fibrotic responses, one might expect to find it in all ERMs, and this was the case in many studies [12,20,21]. However, some investigators also observed a certain number of iERMs apparently lacking collagen I [18,19]. Proteomic profiling confirmed the presence of α1(I) and α2(I) collagen chains [13], and gene expression analysis showed that *COL1A1* gene is expressed by iERM cells [25,26]. Though with some restraints, an attempt to characterize cells responsible for collagen I deposition has recently taken place by confocal microscopy with the identification of glial fibrillary acid protein (GFAP)^-^/vimentin^+^/αSMA^+^/heat shock protein 90^+^ myofibroblast-like cells with collagen I-immunoreactive intracytoplasmic bodies [12]. One shortcoming of this approach, however, is that the collagen I intracellular immunoreactivity can be due to matrix deposition as well as to collagen reabsorption. Previous studies carried out in ERMs from eyes affected by PVR have shown that only a few collagen I-immunoreactive cells are GFAP^+^ elements, for the most part being cytokeratin^+^ epithelial- and fibroblast-like cells or cytokeratin^-^/αSMA^+^ fibroblast-like cells [23,24].

#### 3.1.2. Collagen II

Collagen II is a well-known constituent of the vitreous [1], and it has been reported in iERMs on several occasions [18,19,27]. Nonetheless, its presence does not appear a constant feature, and when detected, collagen II-immunoreactivity occupies a residual part of histologic sections [19,27]. According to Okada et al. [18], however, when iERMs are removed still associated with the ILM, a thin layer of collagen II-immunoreactive ERM can be seen adherent to the ILM. Thus, the presence or absence of collagen II in histologic sections could be related to the removal of the ILM along with the iERM or to the more or less complete detachment of the iERM from the ILM. On the other hand, the more sensitive proteomic approach demonstrated the presence of an α1(II) collagen chain in all tested samples [13].

ERMs from PVR eyes also show an inconstant expression of collagen II which is frequently restricted to small portions of the specimens [23]. Therefore, its presence could be the result of residual fragments of the vitreous cortex trapped within the developing ERMs more than the outcome of novel deposition [18]. This interpretation is corroborated by real-time polymerase chain reaction (RT-PCR) experiments that did not find any relevant *COL2A1* gene expression in PVR ERMs [28].

#### 3.1.3. Collagen III

Collagen III is usually found within collagen I fibrils which are mostly heterotypic fibrils. When the ratio of the two collagens is in favour of collagen III, collagen fibrils assume the aspect of reticular fibres [29], but when the ratio is in favour of collagen I, fibrils are plainly referred to as collagen I fibrils. Although collagen III in iERMs has received little attention, when searched, it was found with the same frequency as collagen I [18,21]. Similar results have been observed in ERMs from PVR eyes [22,23], where the areas occupied by collagen III and collagen I on histologic sections matched [23]. As collagens I and III co-polymerize within the same fibril, the GFAP^−^/vimentin^+^/αSMA^+^/heat shock protein 90^+^ myofibroblast-like cells that are candidate for collagen I production [12] are also reasonably responsible for collagen III deposition. Yet, it is puzzling how proteomic profiling of iERMs did not detect collagen III [13]. On the other hand, in keeping with collagen I/III co-localization within the same fibrils, *COL3A1* was found expressed in ERMs from PVR along with *COL1A1* and *COL1A2* [28].

#### 3.1.4. Collagen IV

**Figure 1:**
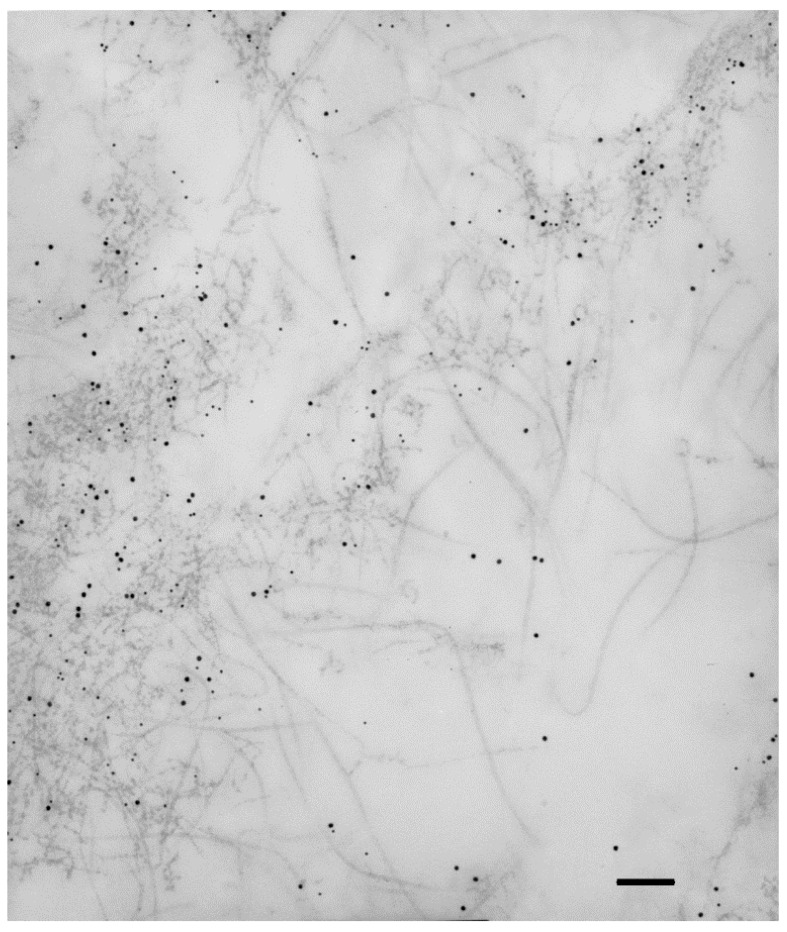
**Collagen IV immunoreactivity is not restricted to basement membranes**. Extracellular matrix of an iERM double-labelled with anti-collagen IV and anti-collagen I antibodies which were unveiled with the appropriate gold-conjugated secondary antibodies. Collagen IV labelling (10 nm gold particles) is restricted to condensations of fuzzy material which is infiltrated by collagen I immunoreactive fibrils (15 nm gold particles). Magnification bar = 180 nm.

Collagen IV is usually considered a basement membrane component [30]. In some instances, collagen IV is also found in interstitial deposits, either in physiologic conditions [31,32], or, more frequently, in fibrotic diseases [33,34]. Collagen IV is a constant constituent of iERMs [12,18,19,20,27], as well as of ERMs from PVR eyes [22,23,35]. A diffuse and considerable intense staining, which does not fit with a mere basement membrane-related immunoreactivity, has been reported by many investigators [12,19,20,21,27]. A study of collagen IV distribution in iERMs has been recently carried out by confocal microscopy showing that this collagen is deposited interstitially, intermingled with collagen I, with a staining intensity that progressively increases towards the vitreous side of the membrane. On the other hand, collagen IV also forms continuous or discontinuous basement membrane-like structures in close association with cells which differ from classic basement membranes by the presence of collagen I [20]. Transmission electron microscopy confirms interstitial condensations of collagen IV-immunoreactive material that are frequently infiltrated by collagen I fibrils (Figure 1). In this respect, collagen IV-immunoreactive areas mostly also co-localize with collagen III and collagen VI [12,20,21]. Anti-collagen IV antibody-labelled material can even be observed within intracytoplasmic vesicles or lysosome-like bodies, suggesting active remodelling of the ECM by ERM cells.

At higher magnification, interstitial collagen IV-immunolabeled condensations often resolve as intricate networks of beaded microfibrils with an interbead distance of 30–40 nm [20]. Collagen IV α-chains occur in six different variants (α1–α6) which assemble to give rise to three collagen IV isoforms: [α1(IV)]_2_α2(IV), α3(IV)α4(IV)α5(IV) and [α5(IV)]_2_α6(IV). So far, the only isoform detected in iERMs by immunohistochemistry is the [α1(IV)]_2_α2(IV) [21]. Proteomic analysis not only corroborates the presence of the α1(IV) and α2(IV) collagen chains but also allowed the detection of α3(IV), α4(IV) and α5(IV) collagen chains [13].

#### 3.1.5. Collagen V

Collagen V is one of the least studied collagens among those detected in ERMs. Actually, to our knowledge, its presence has been assessed by immunohistochemistry only once in ERMs from PVR eyes where it was constantly found [22]. However, collagen V is known to form heterotypic fibrils with collagen I. The ratio between collagen V and collagen I is considered inversely correlated with collagen I fibril diameter [36]. In other words, collagen V is a key regulator of collagen I fibril size: the higher the amount of collagen V, the smaller collagen I fibrils are. On the other hand, the presence of collagen V is not supported by proteomic analysis which did not consistently find COL5A1 and COL5A2 in iERMs [13].

#### 3.1.6. Collagen VI

Collagen VI has been detected in iERMs by several investigators [11,19,20,21]. Its expression has been found particularly relevant only in a subtype of iERMs, the so-called cellophane macular reflex membranes, whereas it is absent or almost negligible in the preretinal macular fibrosis subtype [19]. In contrast to iERMs, collagen VI is not present in ERMs associated with MHs [37]. Collagen VI co-localizes with collagen IV in large areas of iERMs [20]. However, the distribution of the two collagens does not match perfectly as immunoreactive areas for just one of the two collagens are also present [21]. Whereas proteomic profiling of iERMs has not confirmed the presence of collagen VI [13], gene expression analysis did find *COL6A1* mRNA [26].

#### 3.1.7. Collagen VII

A recent addition to the list of collagens associated with iERMs is collagen VII that has been detected in two specimens [38]. However, proteomic analysis did not reveal collagen VII expression in iERMs [13], and RT-PCR did not detect *COL7A1* mRNA in ERM in PVR eyes [28].

#### 3.1.8. Other Collagens

Some additional collagens (IX, XII, XIV, XVIII, XXII) have been identified in iERMs exclusively by proteomics [13]. Collagens IX, XII, XIV and XXII belong to the Fibril-Associated Collagen with Interrupted Triple helices (FACIT) family. Collagen IX is found on the surface of collagen II fibrils, whereas collagens XII and XIV associate with the surface of collagen I fibrils [39]. Thus, even though they have not been specifically investigated, collagen IX should co-localize with collagen II, whereas collagens XII and XIV should match exactly collagen I fibrils distribution. Collagen XXII, in spite of the FACIT molecular structure, is found associated with microfibrils, possibly fibrillin or fibronectin (FN) [40]. It has a very specific pattern of expression, being found at sites of tissue transitions such as hair follicles, myotendinous junctions (skeletal muscles, ciliary body, heart) and muscle insertion to the ribs [40]. It is worth noting that its expression is mostly located where tractional forces are exerted.

Collagen XVIII is a basement membrane-associated collagen [41]. As iERMs contain several basement membrane proteins (see Section 3.2), it is conceivable that collagen XVIII may co-localize with them. Interestingly, collagens IX, XII and XVIII are also proteoglycans as they have GAG side chains. Collagens IX and XII are associated with chondroitin sulfate, whereas almost half the molecular weight of collagen XVIII is due to heparan sulfate side chains [41].

### 3.2. Basement Membrane Proteins and Their Assembly in LNCP Matrix

In addition to collagens IV and XVIII (see Section 3.1), basement membrane proteins include laminins, nidogens/entactins and heparan sulfate proteoglycans.

#### 3.2.1. Laminins

Laminins are trimeric molecules comprising α, β and γ chains. They assemble generating at least 16 different isoforms. Current nomenclature identifies each laminin isoform with a three-digit number which refers to the α, β and γ chains. Thus, for instance, laminin 521 is the isoform composed by laminin chains α5, β2 and γ1. The presence of laminin in iERMs has been frequently reported [19,20,21], and its chain composition has been studied. By immunofluorescence, only laminin α1, β1 and γ1 have been easily detected. Laminin α5 has also been found, though apparently far less represented [21]. Based on these results, iERMs apparently contain laminin 111 and, possibly, minor amounts of laminin 511 [21]. However, proteomic analysis has found a more complex pattern of laminin expression, including α1, α2, α4, α5, β1, β2 and γ1 chains [13]. A wider range of laminin isoforms, therefore, is probably expressed in iERMs.

Even though it can be found alone [20], laminin mostly co-localizes with collagen IV and nidogen/entactin [21]. Co-localization of three major components of basement membranes suggests that the condensations of collagen IV observed in iERMs could be an example of the so-called laminin/nidogen/collagen IV/perlecan (LNCP) matrix (see Section 3.2.4).

#### 3.2.2. Nidogens/Entactins

Nidogen/entactin 1 and nidogen/entactin 2 are proteins typically expressed in basement membranes. They are thought to link laminin and collagen IV lattices together within planar basement membranes [30]. Nidogens/entactins immunolocalization in iERMs found nidogen/entactin 1 co-localized with collagen IV and laminin [21] supporting the existence of LNCP matrix deposits. Proteomic analysis has confirmed the presence of both nidogens/entactins proteins [13].

#### 3.2.3. Heparan Sulfate Proteoglycans

Three heparan sulfate proteoglycans, perlecan, agrin and collagen XVIII, are found in basement membranes [42]. All of them have been convincingly detected by proteomic profiling in iERMs [13]. Heparan sulphate proteoglycans distribution could match LNCP matrix deposits though their immunolocalization has never been assessed.

#### 3.2.4. LNCP Matrix

The LNCP matrix is an extracellular matrix polymer containing laminin, nidogen, collagen IV and perlecan, which does not form planar basement membranes [43]. Though more common in drosophila, examples of LNCP matrix can be found in human tissues as well, including placenta, specifically the foetus–mother interface [32], chondrocyte pericellular matrix [44] and the corneal stroma [31]. As previously reported, laminin, nidogen and collagen IV colocalize in iERMs [21]. Proteomic analysis also identified perlecan in these membranes [13], though its exact distribution has never been assessed. It is possible, therefore, that laminin-, nidogen- and collagen IV-immunoreactive material in iERMs may indeed represent deposits of LNCP matrix. In iERMs, however, collagen IV also co-localizes with collagen I, a feature that likely differs from other examples of LNCP matrix.

### 3.3. Proteoglycans

In addition to being associated with basement membranes, proteoglycans represent fundamental constituents of the ECM ground substance. Yet, they are certainly far less studied than proteins. As a result, investigations on proteoglycans are also extremely limited in ERMs. Perlecan, decorin and tenascin have been detected in PVR-associated ERMs [22,35,45,46]. Opticin has been also observed in some samples of PVR showing a variable pattern of distribution [47]. In iERMs, proteoglycans have been studied only by proteomic profiling. In addition to the previously mentioned heparan-sulfate proteoglycans associated with basement membranes (see Section 3.2.3), a wide variety of proteoglycans including decorin, biglycan, fibromodulin, prolargin (PRELP), opticin, chondroadherin-like protein and podocan have been found [13].

### 3.4. Other Proteins

A few additional proteins have been investigated in the ECM of ERMs. They include microfibrillar, adhesive and matricellular proteins.

#### 3.4.1. Fibrillin

Fibrillin is a microfibrillar protein of the elastic system. It is found as a component of oxytalan, elaunin and elastic fibers. The elastic system of the ECM has been studied by light and transmission electron microscopy only in samples of PVR where oxytalan fibers have been observed either in subretinal or in ERMs [48]. In iERMs, proteomic analysis constantly found fibrillin 1 [13].

#### 3.4.2. Fibronectin

FN is an adhesive protein of the ECM that is expressed in all ERMs, including iERMs [18,19,21,23,35,46,49,50,51]. In iERMs, FN is usually found close to the cell layer (see Section 4) where it partially co-localizes with collagen IV [18,21]. FN can exist as a soluble or a cell-associated form. The latter form differs from the soluble one by the presence of highly conserved alternatively spliced FN type III repeats termed Extra Domain A (EDA) and Extra Domain B (EDB). Both soluble and cellular forms co-exist in PVR-associated ERMs, [52] and the presence of cellular FN has been confirmed by confocal microscope detection of FN EDA. Interestingly, FN EDA immunofluorescence appears in close relationships with myofibroblasts. Alignment of myofibroblast stress fibers with FN fibrils suggests anchorage through fibronexus [7]. Cell-associated FN is also actively produced in iERMs as demonstrated by proteomic analysis and by the presence of EDB FN mRNA [13,25].

#### 3.4.3. Vitronectin

Vitronectin is another adhesive protein of the ECM. Its immunolocalization, to our knowledge, has been studied only in ERMs associated with PVR [49,51], where it is observed pericellularly along with FN, though FN staining appears more diffuse throughout membranes [49]. In iERMs, vitronectin has been detected by proteomic analysis [13].

#### 3.4.4. Thrombospondin 1

Thrombospondin 1 is an ECM protein with multiple functions. It is a matricellular protein and, as such, it is highly expressed upon tissue injury [53]. Thrombospondin 1 has been found expressed only in PVR-associated ERMs [50,54].

#### 3.4.5. Osteonectin/SPARC

An important role in the development of PVR-associated ERMs has been proposed for osteonectin [55]. Osteonectin is a matricellular protein that is also called Secreted Protein Acidic and Rich in Cysteine (SPARC). It has been observed predominantly within cells in retinas affected by PVR [55]. A convincing demonstration of osteonectin in iERMs is lacking, even by proteomics [13].

#### 3.4.6. Periostin

Periostin is another matricellular protein that is highly expressed in PVR-associated ERMs. Its highest expression, however, is found in ERMs associated with PDR. Similar to osteonectin, periostin has been shown within ERM cells [56]. Proteomic profiling did not detect thus protein in iERMs [13].

### 3.5. Glycosaminoglycans (GAG)

GAG constituents of ERMs are a completely uncharted territory. Interestingly, intravitreal injection of chondroitin-6-sulfate triggers the development of experimental ERMs [57]. One might expect that this finding should have prompted researchers to investigate GAG composition of ERMs, either idiopathic or secondary. Surprisingly, this was not the case.

## 4. ECM Arrangement in iERMs

**Figure 2:**
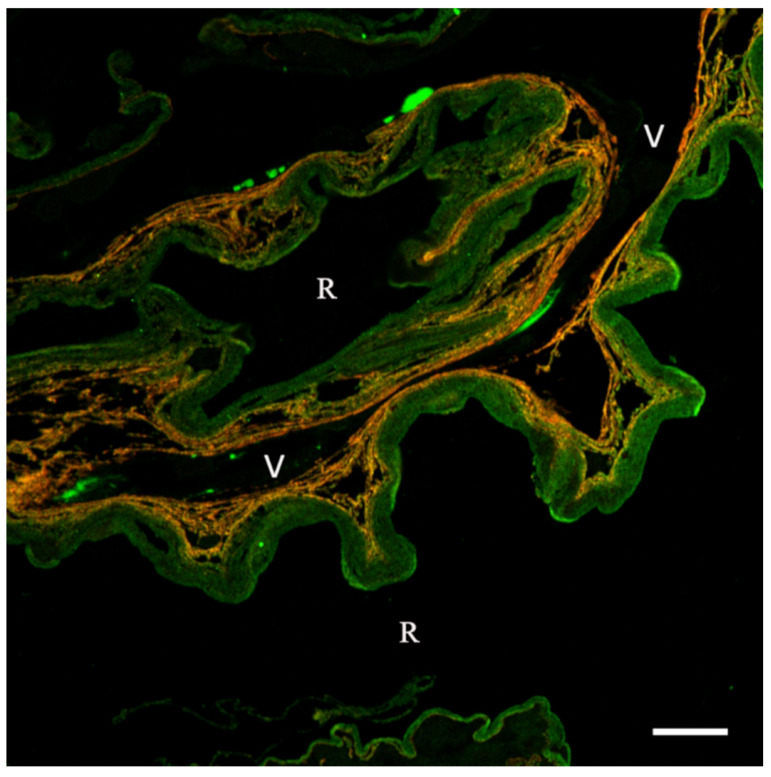
**The iERM lamellar pattern of the ECM as a contributing factor to the tractional properties of the membranes.** Confocal microscopy of an iERM detached from the ILM and labelled with anti-collagen I (green) and anti-collagen IV (red) antibodies. The vitreal (V) and retinal (R) sides of the membrane are indicated. The retinal side of the membrane is exclusively collagen I-immunoreactive, whereas the vitreal side is double labelled. Short strands of double-labelled matrix on the vitreal side of the membrane bridges the extremities of longer segments of the retinal side of the membrane. As a result, the membrane appears producing foldings that can be transferred on the underlying retina if the membrane is adherent enough to the inner limiting membrane. Magnification bar = 20 μm.

The iERMs have an apparent plain bilayered structure consisting of an outer ECM layer, attached to the ILM, and an inner cell layer that faces the vitreous camera. The former layer is composed of a great number of molecules and, despite them seeming randomly deposited, this layer conceals an ordered molecular architecture that a systematic immunohistochemical approach has just started unveiling. Though we still have large gaps of knowledge on iERM ECM composition that wait to be filled (e.g., proteoglycans, GAGs), we already have some information, particularly on collagens, that outline some basic aspects of its arrangement. Few studies have addressed this issue and only recently has an attempt been made by confocal microscopy on multiple-labelled semithin epon-embedded sections that generates images with highly preserved details [12,20,21]. In general, the thickness of the ECM layer is highly variable, having either very thick or very thin regions within the same specimen [21]. With the exceptions of areas thinner than 2–3 μm, iERMs preferentially show a collagen-based lamellar structure (Figure 2) [20,21]. When loosely associated, lamellae can be recognized by light microscopy, whereas when more compactly arranged, their resolution may require electron microscopy [20,58]. With the exception of a thin layer containing collagen II attached to the ILM [18], collagen fibrils are predominantly made of collagen I which apparently spans the entire thickness of iERMs, from the ILM to the cell layer [20,21]. Moreover, collagen VI and III, presenting a distribution very similar to collagen I, possibly span the entire ERM thickness [21]. According to Kritzenberger et al. [19], collagen VI is barely detectable or absent in the preretinal macular fibrosis subtype of iERMs. Though in our experience collagen VI is present in all iERMs [21], a greater amount of collagen VI content in the cellophane macular reflex subtype is supported by Western blotting analysis [19]. Proceeding inward (i.e., towards the vitreous), the [α1(IV)]_2_α2(IV) isoform of collagen IV makes its appearance (Figure 2). A distinct collagen IV layer located just below the cell layer has been previously hypothesized [18]. However, double labelling experiments have demonstrated that collagen IV is intermingled with collagens I (Figure 2), III and VI and that it co-localizes with other basement membrane proteins such as nidogen 1, laminin 111 and possibly minor amounts of an α5 chain-containing laminin [20,21]. Collagen IV immunofluorescence on histologic sections increases progressively towards the cell layer. Finally, just beneath the cells, the ECM appears enriched by an irregular network of FN fibrils [21]. In summary, the sheet of ECM just below the cell layer is presumably formed by collagens I, III, IV and VI, nidogen 1, laminin 111 and FN.

The lamellar arrangement of the ECM and its adhesiveness to the ILM may bear relevance in determining the furrowing of the retinal surface observed in macular packers. It has been observed that ECM lamellae tend to grow shorter, proceeding from the ILM to the cell layer (Figure 2) [21]. Hence, as the ECM is gradually deposited, the inner lamellae, shorter than the outer ones that are attached to the ILM, can exert a progressively stronger tangential traction on the underlying retina. On the other hand, if the ECM layer detaches from the ILM, it can fold on itself under the cell layer creating very thick knot-like arrangements [21]. The adhesiveness to the ILM is likely the result of the interactions between the ECM of the iERM and the very thin inner layer of the ILM made of collagens I and VI along with the [α1(IV)]_2_α2(IV) isoform of collagen IV [12,21].

## 5. An Interactomic Overview of iERM ECM

Mass spectrometry is the technique of choice for reliable and high throughput protein identification from complex protein mixtures. Despite some proteomic analyses having been carried out on vitreous and aqueous samples of eyes affected by iERMs [59,60,61], to our knowledge, the most comprehensive proteomic MS/MS based approach to the definition of iERM protein composition has been carried out only recently [13]. Although we cannot exclude that a number of iERM proteins escaped such analysis [13], because of the applied methods and parameters, the reported proteins can be considered present in iERMs. To shed new light on the pathological mechanisms at the bases of iERM onset and development, we performed a functional revision on iERM protein crosstalks and dynamics that we predicted by building protein–protein interaction networks on a subset of the MS identified proteins [13]. We restricted our analysis to the proteins listed in Table S1 that have been selected according to the criteria described in Section 2. These proteins, retained as occurring in or being correlated to the iERM extracellular environment, may be involved in ECM deposition, composition, organization and dynamics as well as in signaling between iERM ECM and cells. ECM represents a critical framework for cell proliferation, migration and differentiation, not only by providing structural support and molecular signals, but also by controlling the bioavailability of hormones and growth factors as well as the activity of several matrix proteases, their produced matrikines and related inhibitors. Consequently, highlighting the functional correlations existing among iERM ECM proteins and iERM cellular proteins involved in matrix deposition and modulation in cell–cell and cell–matrix interaction and ECM/cell signaling may help us understand the nature and characteristics of the membrane itself.

**Figure 3:**
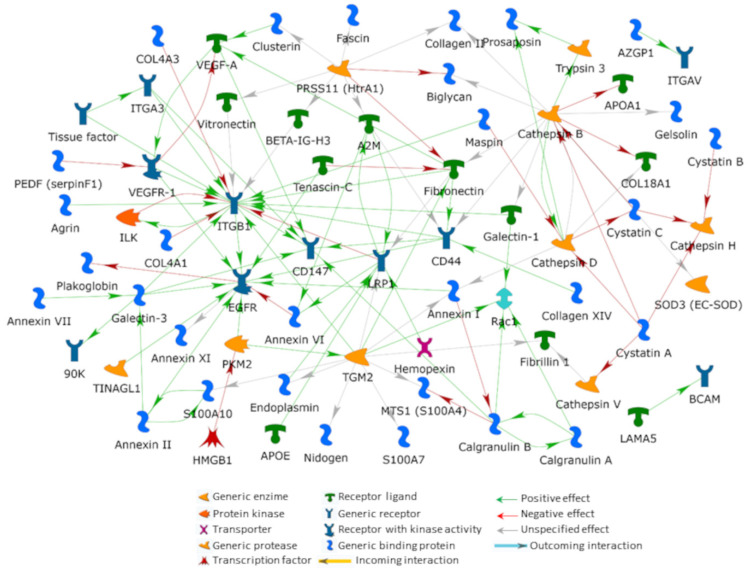
**Direct interaction net**. DIN of 61 proteins present in the ECM of the iERMs and selected from those reported by Christakopoulos et al. [13]. Types of proteins and effects of their interactions are coded according to the legend at the bottom of the figure.

The abovementioned 141 selected proteins were processed by applying the direct interaction and the shortest path algorithms from the MetaCore resource to outline their interactome. According to their direct cross-linking, we generated a net including 43% (61 items) of the investigated proteins (Figure 3). As no additional protein is required for allowing functional interactions among the processed proteins, the DIN visualizes highly probable functional correlations occurring in vivo among the processed factors. Integrin β1 (ITGB1), cathepsin B (CatB), epidermal growth factor receptor (EGFR), protein-glutamine gamma-glutamyltransferase 2 (TGM2) and prolow-density lipoprotein receptor-related protein 1 (LRP1) resulted as the main hubs, with individual relevance corresponding to the order in which they have been listed. While ITGB1, EGFR and LRP1 are transmembrane proteins, CatB and TGM2 are multifunctional proteins that can also be found in the extracellular space and that are involved in ECM modulation and organization as well as in several signal transduction events.

Since they establish most of the outlined net functional connections and in accordance with their previously described functional properties, either in the eye or in other organs, these five hubs can be considered bona fide highly relevant for iERM formation. Although ERMs are referred to as idiopathic when their occurrence is not associated with any other ocular disease, several of the previously identified iERM proteins [13] are intimately involved in different ocular affections, such as trauma, intraocular inflammation, retinal detachment and vascular pathologies, as discussed hereafter.

### 5.1. Integrins

Integrins are the major transmembrane adhesion receptors on the plasmalemma. These heterodimeric proteins (composed by α- and β-subunits) allow cell interaction with the surrounding ECM and transduce tensional forces between the ECM and the cytoskeleton. Such a transduction may involve dozens of factors (i.e., the integrin adhesome) and results in specific kinase or phosphatase activation with related cellular responses, often including modulation in gene expression as well as cytoskeleton and ECM remodeling [62,63,64]. Integrins are known as regulating or intersecting the same signaling pathways of growth factor receptors and playing essential roles in cell–cell interactions and cell–ECM adhesion, such as cell differentiation, proliferation, migration and invasion. For their part, ECM composition and stiffness affect integrin clustering dynamics, thus influencing their capability of forming adhesions and eliciting different cellular responses [65].

In recent years, mechanobiology has come under the spotlight for deciphering the etiopathogenesis of fibroproliferative disorders, and integrins have been recognized as critical receptors for mechanotransduction in chronic inflammation, fibrotic diseases and cancer [66]. Since mechanosignaling is essential in tissue homeostasis, repair and fibrosis [67], it is not surprising that ITGB1, the most frequently occurring β subunit in integrin dimers, is the principal central hub in the DIN from the iERM proteins we selected. This integrin is involved in a plethora of cell–ECM interactions and functions depending on its dimerization with different α-subunits. Besides ITGB1, α_3_ and α_V_ integrins, two usual dimerization partners of ITGB1, have been also identified in iERM [13].

Integrin α_v_β_1_ is an RGD (arginine-glycine-aspartic acid)-binding integrin, a receptor for plasma and ECM proteins such as vitronectin and FN. Highly expressed on activated fibroblasts, α_v_β_1_ integrin plays a relevant role in fibrosis by binding to the latency-associated peptide of transforming growth factor (TGF)-β1 and by mediating its activation [68]. Specific inhibition of α_v_β_1_ integrin attenuates lung and liver fibrosis in the corresponding murine models of the diseases [68]. In addition, α_v_β_1_ integrin has recently been associated with a non-canonical TGF-β signaling pathway that promotes procollagen I synthesis [69]. Integrin α_3_β_1_ mainly mediates cell adhesion to the basement membrane [63], and its signaling is implicated in epithelial phenotype maintenance and physiological wound healing. This integrin dimer is referred to as laminin receptor as it preferentially binds to laminin 5. It also binds to nidogen-1 and to the non-collagenous domain of α3[IV] collagen (α3[IV]NC1) [70,71], both of which have been detected in iERM [13,21]. Interestingly, α_3_β_1_ integrin acts as a negative regulator of collagen IV synthesis and deposition as its lung-targeted downregulation is associated with increased collagen IV expression. Moreover, in a murine model of idiopathic pulmonary fibrosis, α_3_β_1_ downregulation also results in decreased deposition of collagen I and reduced occurrence of myofibroblasts [72].

Activated integrins elicit, intersect and/or share several signaling pathways triggered by different growth factor receptors [73,74]. In this respect, cells in iERM express EGFR and EGFR signaling can also occur in the absence of ligands by an integrin-dependent EGFR phosphorylation mechanism requiring the transient formation of an EGFR-β1 integrin macromolecular complex [74,75]. Indeed, EGFR activation by β1-integrin appears critical for cancer invasion and metastasis through the β1-integrin/EGFR/VEGF-A/VEGFR1 signaling axis in which Rab25 plays a bridging role between EGFR phosphorylation and VEGFR1 activation [76]. Since fibrotic and metastatic processes often share similar signaling pathways, and based on the MetaCore analysis, which highlights a tight functional crosstalk between these receptors and ligands in the DIN (Figure 3), we suggest that a similar functional axis might be also involved in iERM cell precursor migration, survival and proliferation.

#### 5.1.1. Integrins, VEGF and the Avascular Nature of iERMs

In contrast to other ERMs, iERM are avascular. This can be due to the binding partners of α_3_β_1_ integrin that are thought to exert a negative role on angiogenesis acting at several levels [77]. For instance, whereas α_3_β_1_ integrin interaction with α3[IV]NC1 can inhibit angiogenesis via a number of mechanisms, including decreased vascular endothelial growth factor-A (VEGF-A) expression [70], α_3_β_1_ loss in mouse endothelial cells enhances pathological angiogenesis [77]. Despite neovascularization not being found in iERM, VEGF-A is one of the main central hubs in the MetaCore SPN (Figure S1), suggesting a relevant role played by this factor in iERM pathogenesis. Different eye injuries may unleash inflammation and immune responses leading to VEGF-A upregulation. Hyalocytes and Müller cells, both capable of transdifferentiating into myofibroblasts and considered main sources of iERM cell components [10,78], can produce and secrete VEGF-A [79,80]. Along with retinal pigment epithelial (RPE) cells, which may contribute to iERM cell population as well [81], Müller cells are also active in angiogenic homeostasis by releasing pigment epithelium-derived factor [82], a neuroprotective factor showing potent anti-inflammatory and anti-angiogenetic roles that counteract VEGF-A [83].

Another mechanism that impairs iERM neovascularization, in spite of VEGF-A expression, possibly relies on the negative regulatory role assigned to VEGF receptor (VEGFR)1, the VEGF receptor type identified in iERMs [13]. Cell surface VEGFR, and its soluble counterpart are highly competitive inhibitors of the VEGFR2, which operates as a VEGF-A pro-angiogenetic receptor [84]. Although multiple cell types could release VEGF-A during iERM formation and development, the presence of VEGFR1 may reduce levels of free VEGF-A, preventing its binding to VEGFR2 and/or modulating VEGFR2 signaling by the formation of VEGFR1/VEGFR2 heterodimers [85,86]. On the other hand, VEGF-A binding to VEGFR2 could also be inhibited by endostatin, an anti-angiogenic fragment of collagen XVIII that is released by cathepsins B, L, V and K action [87,88]. Interestingly, collagen XVIII as well as cathepsins B, L and V have been found in iERM [13].

### 5.2. Epidermal Growth Factor Receptor (EGFR)

The EGFR, also known as ErbB1/HER1, is a transmembrane glycosylated protein receptor with tyrosine kinase activity. EGFR is one of the main hubs in the DIN outlined in Figure 3. Almost all cell types express receptors belonging to the EGFR-family. These receptors, highly expressed in cancer cells [89], deeply affect cell fate, being implied in cell proliferation, survival, epithelial-to-mesenchymal transition (EMT), migration and invasion. Recently, its direct interaction with pyruvate kinase (PKM2 in Figure 3) and its nuclear localization were proved, leading to the expression of genes involved in developing a stem cell-like phenotype, thus supporting invasion and metastasis [90]. EGFR was also detected in lung myofibroblastic foci of patients affected by idiopathic pulmonary fibrosis [91], and its aberrant prolonged signaling was associated with increased expression of TGF-β1, interstitial myofibroblast proliferation and progressive fibrotic injury in the kidney [92]. In the eye, RPE cells can produce epidermal growth factor (EGF), which determines autocrine-induced proliferation and migration via the EGF/EGFR/MAPK signaling pathway. Inhibition of this pathway may reduce the development of PVR, and targeting EGFR has been proposed as a therapeutic strategy for preventing PVR formation [93,94]. In view of its ability to induce migration and EMT, TGF-α, another EGFR-binding member of the EGF family, has also been suggested to play a role in PVR formation [95]. Interestingly, both TGF-α and VEGF-A were found significantly upregulated in the vitreous of PVR patients [95], hence supporting once more, a relevant role that the EGFR/VEGF-A/VEGFR-1 signaling may exert on the development of some forms of ERMs. As we have already noted, the part played by the EGFR might also be independent from ligand engagement as it can be activated by the interaction with β1 integrin [74,75].

### 5.3. Galectin-3 Lattices

Galectin-3 is not one of the main hubs of the DIN shown in Figure 3. However, its presence tightens the functional relationship between ITGB1, VEGFR and EGFR. This β-galactoside-specific lectin is a cytoplasmic and nuclear protein secreted by various cells in the extracellular space. Here, according to its concentration and environmental conditions, galectin-3 oligomerizes and binds specific glycosylated ligands on the cell surface, thus forming galectin lattices [96]. Such cell surface molecular lattices are dynamic microdomains fundamental in tuning the function of several receptors, including EGFR, VEGFR, fibroblast growth factor (FGF) receptor, platelet-derived growth factor (PDGF) receptor and TGF-β receptor, and in controlling their lateral diffusion, clusterization, signaling, endocytosis and recycling [96,97]. Its binding to glycoproteins associated with the cell membrane (e.g., FN) and its integrin-binding and clustering induction promote integrin-dependent FN fibrillogenesis and cell adhesion [96,98]. Notably, galectin-3 binding to α_v_β_1_ and, to a lesser extent, to α_3_β_1_ integrins has been recently reported mediating cell adhesion to the substrate [98]. On the other hand, galectin-3 may also stimulate integrin endocytosis and focal adhesion turnover, thus reducing cell anchorage to the ECM and affecting related signaling [96,98].

In the attempt to define the biochemical bases of iERM pathogenesis, the acknowledgement of galectin-3 as a central regulator of inflammation and fibrosis is of great relevance [99,100]. Highly expressed in fibrotic tissue from different organs [101,102,103,104], galectin-3 has been suggested working even as a toll-like receptor (TLR) 4 ligand [105] and as a damage associated molecular pattern protein [106].

Galectin-3 also induces the clustering and activation of the highly glycosylated transmembrane protein CD147 notoriously involved in several inflammatory and fibrotic disorders [107,108,109]. Through CD147, also referred to as extracellular matrix metalloproteinase inducer (EMMPRIN/basigin), galectin-3 is indeed thought to induce expression of metalloproteinases and to concur to myofibroblast differentiation [108,110]. CD147, expressed at significant levels by RPE cells, astrocytes and Müller cells [111,112,113], has been found in iERM [13]. In DIN (Figure 3), CD147 acts as a molecular bridge for the connection of galectin-3 with CD44 and VEGF-A, thus stressing the relevance that galectin lattices may have in controlling several ECM/growth-factor/membrane receptor signaling pathways during iERM formation and development. In particular, CD147 co-localization with CD44, possibly mediated by galectin lattices, has been recently reported crucial in establishing proper mechanical strength for stress-fiber deposition and myofibroblast differentiation [107]. Last but not least, CD147 may concur to the formation of iERM, by a galectin-3-induced co-clustering with β_1_ and α_3_ integrins as previously suggested for PVR [113].

### 5.4. CD44 Antigen

As we have already noted, ITGB1 is a key receptor of various matrix proteins and a critical modulator of several receptors for signal transduction. In addition to EGFR and VEGFR-1, ITGB1 directly binds or functionally correlates to CD44 antigen. Based on DIN and SPN, CD44 is likely a critical factor in iERM dynamics. MetaCore recognized a relevant role of this protein in the SPN. Here, CD44 results in the second main hub of the net (Figure S2). In addition, it establishes direct interactions with four of the eight central hubs from DIN and/or SPN (i.e., ITGB1, EGFR, FN and CatB).

CD44 is a single-pass transmembrane matrix adhesion receptor for hyaluronic acid (HA) [114]. It serves as a mechanosensor and controls cell adhesion and proliferation, ECM synthesis, deposition and degradation, and recently it has been also associated with chondrocyte dedifferentiation in response to excessive mechanical stress loading [115]. In the retina, CD44 is expressed by Müller cells [116] and astrocytes [117]. In accordance with histopathological studies, reporting glial cells as one of the main ERM cell types [118], CD44 presence has been strongly correlated to the development of ERM, where it has also been described on the cell membrane of myofibroblasts and macrophages [28,119,120]. In non-resolving inflammatory events, both FN EDA isoform and HA act as matrix-derived damage-associated molecular patterns and engage, besides TLR4, CD44 and integrins. Related signaling, in combination with the TGF-β pathway, affects the wound healing program and may lead to fibrotic responses and chronic diseases [121]. CD44 has been described controlling the ECM HA-composition by mediating endocytosis of small oligosaccharides derived by the hyaluronidase 2-dependent HA-degradation. As HA controls, depending on its molecular weight, several properties and functions of the ECM as well as tissue inflammation, CD44 may be crucial in ERM development. When bound to HA, CD44 is in fact implicated in modulating cytoskeleton organization, focal adhesion functionalities and proliferative induction by acting as a co-receptor of TLR2/4 and EGFR [121]. Despite many cell types in the eye exposing TLRs on their surface [122,123], to our knowledge, TLR2/4 proteins have not been described in iERM. Therefore, it is possible that in iERM, HA-CD44 may mainly engage EGFR with consequent MAPK/ERK signaling, α-SMA expression and regulation of myofibroblast differentiation [124]. As mentioned above, CD44 may further support myofibroblast differentiation depending on TGF-β1 stimulation and CD147 mediation [107]. CD44 is known as interacting with CD74, also referred to as the MHC class II-associated invariant chain, thus concurring to macrophage migration inhibitory factor (MIF) signaling [125], a cytokine classically associated with disorders having an inflammatory component, including proliferative retinopathies. The MIF/CD74/CD44 complex controls, through Src activation, the PI3K/NF-κB and ERK/NF-κB pathways. In this way, CD44 may affect gene expression of pro-inflammatory cytokines and cell proliferation [126]. Although CD74, classically reported as the MIF receptor, has been not identified in iERM [13], MIF signaling may occur even in its absence [127,128]. Indeed, similar to PDR [129], an active MIF/CD74/CD44 pathway might be in place even in iERM where MIF has been detected as well [13].

In summary, CD44 may influence iERM development and ECM deposition through a very large number of interactions that can activate several signaling pathways.

### 5.5. ECM Synthesis and Degradation Loop

#### 5.5.1. Cathepsins

Based on the GO-term enrichment analysis performed by the DAVID resource to highlight the most relevant terms annotating the iERM MS-protein list, the involvement of both endo- and extracellular proteolytic events appear to play a key role in ERM pathophysiology (43 proteins shared the Biological Process GO-term “proteolysis”; *p* value = 1.2 × 10^−7^). After all, ERM formation and development are referred to a fibrotic process characterized by myofibroblast transdifferentiation, aberrant migration and proliferation, by excessive ECM production and proteolytic degradation and, finally, by myofibroblast cytoskeletal contraction [78].

In the DIN (Figure 3), the extracellular proteolytic aspect of iERM is apparently centered on CatB. The tight crosstalk between CatB and cathepsin D, as well as their correlation with previously identified other cathepsins (i.e., cathepsins H and V) and cathepsin inhibitors (i.e., cystatins C, B and A) [13], significantly stresses the relevance that these key signaling molecules may assume in the disorder [130]. Their sub-net may be related to an aberrant control in protease/protease-inhibitor balance, with possible shedding of several signaling factors associated with inflammatory cell infiltration, to the activation of TLRs recognizing pathogen- and damage-associated molecular pattern molecules [131], to NLRP3 inflammasome triggering [132] and to antigen processing and presentation [133]. These considerations are in line with the hypothesis that inflammation and reactive oxygen species-related injury may be critical processes in ERM formation [134,135], as also suggested by the significantly enriched DAVID GO-term “response to reactive oxygen species” protein cluster (including 15 proteins from the list of identified proteins provided in reference 13; *p* value = 2.5 × 10^−11^). Interestingly, cathepsin release in the cytosol is related to lysosome membrane permeabilization, a process caused by several different stimuli, including oxidative stress associated with ionizing radiations [136].

CatB is a lysosomal cysteine proteinase whose overexpression has been widely associated with inflammation, collagen deposition and fibrosis. CatB, which is highly expressed by microglial cells and macrophages, promotes ECM degradation by directly participating to its proteolysis [137], by activating metalloproteinases [138] and by eliminating the tissue inhibitors of metalloproteinase (TIMPs) [139]. ECM and basal membrane degradation by cathepsins and metalloproteinases also leads to the release of bioactive matrix fragments, the matrikines, that are implicated in numerous biological processes including wound healing and fibrosis [140,141]. As mentioned above (see Section 5.1.1), CatB and cathepsin V can play an anti-angiogenic role by releasing the matrikine endostatin from collagen XVIII.

Mesotrypsinogen is another CatB target found in iERM [13]. Its activation to trypsin 3 [142] may further contribute to ECM proteolysis and iERM cell precursor migration or even concur in unbalancing the ratio between proteases and their cognate anti-proteases. Trypsin 3, along with caspase-14, the only caspase identified in iERM by the MS-based proteomic [13], is also involved in prosaposin processing and generation of saposins A–D. Whereas extracellular prosaposin is a neurotrophic factor highly expressed and secreted by RPE [143], saposins A–D are lysosomal proteins active in the catabolism of glycosphingolipids. Saposin B, associated with defective RPE secretory proteostasis [143], has been implicated in age-related macular degeneration [144]. Though a direct involvement of saposins in iERM development to our knowledge has never been reported, the co-expression of mesotrypsinogen and caspase-14 supports the extracellular generation of saposins with still unknown effects on ECM proteins.

#### 5.5.2. Protein-Glutamine Gamma-Glutamyltransferase 2 (TGM2)

As previously reported in several fibrotic diseases, CatB may contribute to myofibroblast transdifferentiation by activating TGF-β1 [145,146,147]. Among other things, TGF-β promotes ECM protein deposition and cross-linking into proteolytic resistant complexes by inducing TGM2 [148,149]. Not surprisingly, TGM2, also referred to as transglutaminase 2 or tissue transglutaminase, is a central hub in DIN (Figure 3) where it is functionally associated with FN and ITGB1. TGM2 not only enzymatically cross-links FN, but also acts as an integrin-binding adhesion co-receptor for FN itself and even promotes the assembly of the latter by interacting with the α_5_β_1_ integrin [150]. TGM2 is a matrix stabilizer whose upregulation has been observed in several disorders characterized by increased ECM protein cross-linking and matrix accumulation. It has been reported as causative for a number of fibrotic diseases, even in the eye [148,151], and possibly necessary to tissue inflammation stabilization [149]. In addition to FN, TGM2 targets several other ECM proteins, such as collagen, fibrinogen, laminin, vitronectin, nidogen and fibrillin-1, all identified in iERM [12,13,21]. In particular, TGM2 stabilizes interactions between laminin and nidogen [152], as well as between FN and collagen [153]. Obviously, dyshomeostasis in inter- and intra-molecular cross-linking of such proteins, as well as in their aminylation with biogenic amines/polyamines, affects their structure, solubility and function and may affect related mechanosignaling. Interestingly, TGM2 may influence mechanosignaling even intracellularly by targeting cytoskeletal proteins, including actin, tubulin, and myosin [154]. TGM2 is induced by EGF, UV light and viral infections [154]. However, TGM2 expression and function depend on its redox state [155,156]. Indeed, TGF-β-induced TGM2 activation was reported as a reactive oxygen species-dependent process, and its upregulation in astrocyte cultures is caused by oxidative-stress upon glutamate prolonged exposure [157]. In allergic inflammation, TGM2 is capable of controlling reactive oxygen species production as well as histone deacetylase-3 and snail expression, by interacting with rac1 and NF-κB, respectively [158]. Of relevance, the transcription factor snail, an E-cadherin suppressor, is a crucial regulator of EMT and glial-to-mesenchymal transition [78,159]. In this respect, snail is upregulated by TGF-β1 in iERM Müller cells undergoing myofibroblastic transdifferentiation [78] and in RPE cells during EMT in PVR [160].

#### 5.5.3. High-Temperature Requirement A Serine Peptidase 1 (HTRA1)

In parallel to cathepsins, the high-temperature requirement A serine peptidase 1 (HTRA1) (PRSS11 in DIN, Figure 3) is active on some iERM proteins, represented in DIN by FN, vitronectin, collagen II, biglycan and α2-macroglobuline. In view of its four protein domains (i.e., insulin-like growth factor binding domain, kazal-type motif, trypsin-like peptidase proteolytic domain and PDZ domain), this member of the HTRA family of serine proteases is a multifunctional protein. HTRA1 is active in several contexts. Specific to fibrotic diseases and/or to the eye, HTRA1 is involved in signal transduction inhibition of TGF-β family proteins [161], in keloid lesion formation [162], in retinal neovascularization [163], and even in mediating injury in RPE exposed to short-wavelength light [164].

HTRA1-dependent ECM degradation controls the bioavailability of some growth factors including FGF and insulin-like growth factor [165,166]. The latter has been implicated in ERM progression and contraction [167], and it was found in iERMs as well [26]. FGF, too, has been found expressed in iERM [26,168], where it may participate to membrane formation by supporting survival and proliferation of glial cells [135] and the activation and dedifferentiation of RPE cells [169].

#### 5.5.4. Prolow-Density Lipoprotein Receptor-Related Protein 1 (LRP1)

Despite the outlined net of degrading enzymes, ECM is known to increase in ERM. Two fine regulators of many pathways that can lead to the accumulation of the ECM in iERM are represented by the LRP1, one of the central hubs in DIN (Figure 3), and by its main target protein, the wide-spectrum proteinase inhibitor α2-macroglobulin (A2M) [170]. They may modulate the TGM2-induced ECM stabilization and the proteolysis operated by several of the iERM identified proteases, as well as the ITGB1-dependent fibrogenic-signal transduction [170]. LRP1 is a ubiquitous highly versatile protein that, because of its capability of interacting with more than 100 different extracellular ligands and with several intracellular proteins, acts a fine signal pathway integration in several contexts including cell proliferation, inflammation, wound healing and tissue repair [171,172]. Its functions span from lipid and glucose metabolism to the modulation of ECM synthesis and turnover [171]. It also includes regulation of cell membrane protein composition and related signal transduction events [173], control of extracellular proteases and lysosomal enzymes activation [174]. Regarding the built DIN (Figure 3), LRP1 can promote ITGB1 maturation and transport to the cell surface [175] and its internalization and degradation [173,176]. LRP1 also stimulates internalization and degradation of TGM2 [177], and serpin:enzyme or protease activated A2M complexes (i.e., A2M: protease complexes or simply A2M*) [174]. Consequently, defects in LRP1 interactions with TGM2, ITGB1 or serpin:enzyme/A2M: protease complexes result in ECM and signal transduction aberrances with cell adhesion and migration defects. Nonetheless, as different cell types and related microenvironments in distinct physiological and pathological conditions have diverse ligands and intracellular proteins interacting with LRP1, the protective or pathogenic roles played by this receptor are cell type-, time-, and tissue-dependent [178]. During lung repair, LRP1 was suggested to allow tissue repair, also limiting inflammation and fibrogenetic risks. Prolonged LRP1 downregulation or deficiency leads to TGF-β1 release and to impaired TGM2 and metalloproteinase clearance with consequent excessive ECM deposition, proteolysis cross-linking, increased periostin synthesis, myofibroblast contraction and fibrotic lesion formation [172,179]. Moreover, LRP1 may control ECM stabilization by mediating FN catabolism and reducing its accumulation. On the other hand, the interaction between A2M* and LRP1 induces Müller cell migration by controlling metalloproteinases activity [180]. In addition, A2M may indirectly promote proliferation of migrated ERM precursor cells by inhibiting plasmin- and kallikrein-induced fibrin polymers digestion. Indeed, it has been suggested that the matrix formed by fibrin polymers in the vitreous allows proliferation of the surrounding cells [181]. In line with such a hypothesis, increased fibrinogen levels have been detected in the vitreous from patients affected by idiopathic MHs [182] or iERM [59].

Since it acts as a carrier molecule, A2M may also control local concentrations of several growth factors and cytokines (i.e., PDGF, FGF2, TGF-β, insulin and IL-1β) with possible consequences in cell proliferation, fibrinogenic signaling and inflammation. Interestingly, A2M* has been very recently recognized as the signaling ligand for the 78 kDa glucose-regulated protein cell surface chaperone (GRP78/BiP) in diabetic nephropathy. A2M* binding to GRP78 promotes the activation of the profibrotic Akt pathway, thus inducing the production of matrix proteins and profibrotic cytokines [183]. Although GRP78 cell surface expression is mostly due to high glucose stimulation, we cannot exclude a priori a similar localization in iERM where this chaperone has also been identified [13].

The occurrence of A2M as well as of the non-inhibitory serpin maspin [184] in the DIN (Figure 3) underlines the presence of a protease-inhibitory system, parallel to the above described cystatin-centered one, downregulating ECM proteolysis in iERM. The involvement of A2M in ERM development is further stressed by its capability to induce CD44 and ITGB1 expression [185]. Hence, it is quite evident how a defective crosstalk between LRP1 and A2M may have detrimental effects on iERM formation and development.

## 6. Fibrogenic Biomarkers in the Vitreous Humor: Growth Factors, Cytokines, mRNA and miRNA

The assessment of molecule concentrations in the vitreous humor (VH) represents a good quantitative method to evaluate the involvement of soluble and secreted molecules in the etiopathology and progression of iERMs. Studies along this line are not numerous, and even though the proteins found in the VH are not necessarily produced by iERM cells, they may certainly interact and influence iERM development, with even more significance if they are not found in the VH of control groups. Since MS-based proteomic investigation may have missed the identification of several soluble proteins in iERMs [13], we also included in this review studies that quantified VH cytokines and growth factors that are known to be involved in fibrogenic responses.

By enzyme-linked immunosorbent assay, several molecules have been found upregulated in the VH of eyes with iERM, and accordingly, they have been proposed as iERM promoting factors for migration and/or proliferation of cells. Among the fibrogenic factors, TGF-β is certainly the most powerful. In line with the observations that TGF-β2 is the main TGF-β family member expressed in the eye [186,187], levels of TGF-β2 have been found significantly increased in VH samples from eyes with iERM compared to control groups [188,189]. Furthermore, a positive correlation has been found in the vitreous fluids between the levels of TGF-β2 and the soluble form of the low-density lipoprotein receptor-relative with 11 ligand-binding repeats (LR11) [188]. LR11 is a type I membrane protein that plays a key role in the migration of immature vascular smooth muscle cells [190]. Soluble LR11 (sLR11) is released from the cell surface by proteolytic shedding and its levels in VH and sera are increased in diabetic patients either developing or non-developing PDR [191]. Interestingly, sLR11 has also been described to inhibit the activation of the signaling pathway downstream of the bone morphogenetic protein-7, a member of the TGF-β receptor superfamily with antifibrotic activity that antagonizes TGF-β2-mediated PVR development [192]. Thus, even though the site of sLR11 origin has not been identified yet, it has been speculated that vitreous sLR11, in association with TGF-β2 signals, may cooperate in promoting the pathological migration and proliferation of cells concurring to the formation of iERMs [188]. Moreover, sLR11 may also potentiate TGF-β2-induced fibrogenesis by blocking the signaling of molecules playing antagonistic roles. However, the level of TGF-β2 in VH from patients with iERMs is still a controversial issue. A survey of 43 growth factors and chemokines by multiplex beads analysis in the VH of a large group of patients (31 with iERMs and 30 with idiopathic MHs as control group) did not demonstrate any significant increase of TGF-β1, TGF-β2 or TGF-β3 [193]. In contrast, levels of 31 cytokines (CCL21, CXCL13, CCL27, CXCL5, CCL24, CCL26, CCL11, CCL6, CXCL1, CXCL2, CCL1, IFN-c, IL-10, IL-16, IL-1b, IL-2, IL-4, IL-6, IL-8, CXCL11, CCL8, CCL7, CCL13, CCL22, CXCL9, CCL3, CCL19, CCL23, CXCL12, CCL25 and TNF-α) were higher in the VH of eyes with iERM, a result compatible with the activation of fibrotic and inflammatory processes. Levels of TGF-β1 in the VH, on the other hand, seem to be unaffected by the presence of iERMs [189,193,194]. This does not mean that TGF-β1 has no role in iERM development as its expression was detected within iERMs [25,195]. TGF-β1 and TGF-β2 influence iERM progression and matrix deposition as at least a subpopulation of ERM cells express the TGF-β receptor. In particular, the TGF-β receptor 2 subunit with kinase properties has been demonstrated in iERMs cells [7], mostly in GFAP^−^/vimentin^+^/αSMA^+^ myofibroblast-like cells that also express heat shock protein 90 [12].

PDGF-AA is the only protein among a battery of 29 growth factors/cytokines tested by multiplex protein array that has been found at a considerable higher level in the VH than in the aqueous humor of patients with iERMs [193]. Gene expression analysis has also shown the presence of PDGFB mRNA in iERMs [26], as if to say that PDGF-AB and/or PDGF-BB can also be produced by iERM cells. Whereas PDGF-AA was described as a potent fibroblast mitogen [196], PDGF-AB isoform has been recently proved to promote collagens I, III and VI deposition in an in vitro model of dermal fibroblasts [197]. To our knowledge, expression of the PDGF receptor has never been investigated in iERMs. On the other hand, both PDGF and its receptor have been frequently detected in other types of ERMs [198,199].

Nerve growth factor (NGF) is another growth factor that has been found increased in the VH of patients affected by iERMs with respect to control eyes [189]. NGF is a small protein that binds to and induces dimerization of tropomyosin receptor kinase A (TrkA) and p75 neurotrophin receptor (p75^NTR^). In addition to its well-known neurotrophic action, NGF has been more recently associated with pro-fibrogenic responses [200]. In particular, NGF promotes myofibroblast differentiation [200,201], inducing α-SMA expression and collagen I and III synthesis and deposition [201,202,203]. As both NGF and its receptor have been found expressed in iERM myofibroblasts [194], their involvement in the deposition of membrane ECM is plausible, though the relevance in the overall process is still undetermined.

In spite of its repeated detection in iERMs [13,26,195,204], VEGF-A has not been found in the VH of iERM patients [59,205,206]. These apparently contrasting results can be explained by the avascular nature of iERMs that, conversely to neovascular ERMs [207,208], may easily correlate with low or undetectable levels of VEGF-A in the VH. On the other hand, as discussed above, VEGF-A can be produced by Müller cells [80], which also express its primary signaling receptor VEGFR2, suggesting the existence of an autocrine role for VEGF-A in iERM Müller cell survival, as previously demonstrated in the adult mouse retina [209].

The example of VEGF-A is emblematic of how studies carried out on the VH should not be used as an indirect way to study iERMs protein expression. To this aim, they should be contextualized and corroborated by direct findings on the membranes. Indeed, the observation that the VEGF-A mRNA extracted from irrigation solution samples collected during vitrectomies from eyes with iERM is increased compared to samples from eyes with MHs [210] supports the notion that VEGF-A is produced within iERMs though it does not diffuse to the VH in detectable amounts.

As just observed, RNA molecules present in the VH can be informative. However, the same caution expressed for results obtained from enzyme-linked immunosorbent assay tests on the VH should be applied for quantitative RT-PCR as well. Indeed, the source of mRNA molecules may be other than just iERM cells. Gene expression analysis, performed through quantitative real time polymerase chain reaction, confirmed some findings previously described, including TGF-β2 and NGF upregulation [194,210]. In the VH of iERM Myojin and coworkers also assessed upregulation of IL-6, CXCL1 and the NF-κB subunit RELA [210], all related to aging and inflammation. However, the actual source of these molecules in eyes with iERMs is still unknown.

RNA molecules retrievable from VH are not limited to mRNA but also comprise miRNAs, as recently also shown in patients affected by iERMs. Five miRNAs appear differentially expressed in iERM VH with respect to the VH recovered by control subjects affected by MHs: four upregulated (miR-19b, miR-24, miR-155 and miR-451) and one downregulated (miR-29a) [211]. Interestingly, miRNA-19b and miRNA-24 have been reported to modulate TGF-β signaling, indirectly supporting a correlation between TGF-β expression and iERM development and suggesting a significant role for these miRNAs in TGF-β-mediated fibrogenesis [211,212,213,214,215].

## 7. Translational Medicine in iERM: The Pharmacologic Vitreolysis

At present, there are limited therapeutic options for iERM. Because the majority of iERMs remain relatively stable over time [216], watchful waiting seems a reasonable choice in cases with absent/mild iERM-associated visual impairment. Surgery represents the elective therapeutic approach at disease stages in which patients experience serious visual symptoms. The standard intervention in these cases consists of mechanical removal of the fibrocellular tissue from the surface of the inner retina via pars plana vitrectomy, a procedure that is supposed to improve post-operative vision quality by relieving the abnormal tractional forces that distort retinal architecture. However, while the beneficial effect of surgery in case of severe disabling iERM is widely accepted, functional outcomes are still largely unpredictable based on the pre-operative characteristics of affected eyes, and the value of surgery remains uncertain in case of mildly symptomatic iERM [217]. Additionally, as with any intraocular surgical procedure, this surgery carries risks of harm from side effects [218].

Pharmacologic vitreolysis is a procedure consisting of the use of exogenous agents to alter the biochemical and biophysical states of the macromolecules responsible for maintaining vitreous structure and vitreo–retinal adhesion [219]. Because dynamics of vitreous separation from the inner retinal surface play a crucial role in the pathophysiology of relatively common vision-threatening conditions such as iERM and disorders of the vitreoretinal interface (i.e., vitreo–macular traction with or without associated full-thickness MH), the intravitreal use of vitreolytic pharmacological agents is of clinical interest as it may represent a minimally invasive option to prevent or treat these conditions. Research efforts on this ground led to the approval by international public health agencies of ocriplasmin, the first vitreolytic agent introduced into clinical practice to treat vitreoretinal interface disorders. The approval of this molecule was achieved based on the results of the Microplasmin for Intravitreous Injection–Traction Release without Surgical Treatment (MIVI-TRUST) studies [220,221], two double-blind randomized controlled trials having vitreo–macular adhesion resolution 28 days after injection, without the creation of an anatomical defect (including MH) or the need for vitrectomy, as the primary end point.

In addition to this specific field of application, it has been postulated that pharmacologic vitreolysis may have a beneficial role as an adjuvant factor in the surgical treatment of iERMs. In fact, the use of vitreolytic agents might facilitate surgical maneuvers of fibrocellular membrane cleavage by interfering with the adhesion system between the epiretinal fibrocellular components and the inner surface of the retina. However, it is noteworthy that the clinical value of pharmacologic vitreolysis in the treatment of iERM has not yet been elucidated. Quite the opposite, the efficacy of pharmacologic agents in releasing vitreoretinal traction has been reported as inferior in case of iERM presence [222]. In this respect, a detailed knowledge of the iERM and internal limiting membrane composition is essential to identify appropriate targets of pharmacologic interventions and to design new classes of vitreolytic agents that may also act on iERM. Some authors described a layer of native vitreal collagen (type II) between the fibrocellular proliferation and the ILM [18] and surmised that plasmin or other vitreolytic enzymes might have the potential to degrade it [223]. Several enzymes have been proposed by other groups to partially hydrolyze the ECM of iERMs, possibly facilitating iERM peeling and the associated vitrectomy [224,225].

The main constituents of the ILM are collagens IV, VI and XVIII, laminin, fibronectin, proteoglycans, entactin and possibly hyaluronan [226,227,228,229]. A recent analysis of laminins and collagen chain expression provided more detailed evidence that the ILM specifically contains laminin 111, laminin 521, laminin 211, collagens I and VI and the [α1(IV)]_2_α2(IV) and α3(IV)α4(IV)α5 isoforms of collagen IV [21]. However, molecules facing the vitreal side of the ILM that can be more easily reached by vitreolytic agents are collagens I, VI and collagen [α1(IV)]_2_α2(IV). Laminins, entactin and collagen α3(IV)α4(IV)α5, on the other hand, are concentrated on the retinal side [21,230]. As we have already noted, the components of the ECM of iERMs are numerous and create an important network of interactions. Each of these components could be considered a potential pharmacological objective. However, attacking the main iERM structural components (laminins, collagens and fibronectin), or the bindings between iERM and ILM, has been the first and the only strategy attempted so far.

Without the claim of providing an exhaustive list and accurate descriptions for which dedicated and excellent reviews can be found [231,232,233,234], we hereby briefly report the vitreolytic molecules that have been studied to date, with special focus on agents targeting the structural components of the membranes or their adhesion to the ILM.

Vitreolytic agents can be classified based on their biologic action or chemical activity [235]. Posterior vitreous detachment (PVD) results from concurrent liquefaction of the vitreous body and weakening of vitreoretinal adhesions. Those agents that primarily liquefy the vitreous gel are referred to as liquefactants, while those that induce dehiscence at the vitreoretinal interface are called interfactants. Several agents are considered to have both liquefactant and interfactant effects, a favourable characteristic given that these actions should be applied simultaneously to obtain an efficient and safe pharmacologic PVD induction. With regards to their chemical properties, vitreolytic agents may be enzymatic or non-enzymatic. Within the larger enzymatic group of agents, a further subdivision can be made based on the substrate-specific/nonspecific action of each agent.

### 7.1. Collagenase

Collagenase from *Clostridium histolytica* has been employed because of its ability to cleave collagen II inducing vitreous liquefaction [236]. Important side effects due to its unrestricted proteolytic activity induced us to drop the experimentation of this vitreolytic agent.

### 7.2. Hyaluronidase

Hyaluronidase is a substrate-specific enzymatic agent that targets HA, a GAG composed of repeating disaccharide units of *N*-acetyl glucosamine glycosidically linked to glucuronic acid. Hyaluronidase is a liquefactant agent and does not induce vitreoretinal dehiscence and PVD. Preclinical studies demonstrated efficacy of hyaluronidase in animal models. Phase III trials aimed to assess its superiority in accelerating vitreous hemorrhage clearance compared to saline solution supported a therapeutic utility of ovine hyaluronidase but did not provide sufficient evidence to warrant approval as a commercial product [237].

### 7.3. Chondroitinase

Chondroitinase is another substrate-specific enzyme that cleaves and fragments chondroitin-sulfate-containing GAG side chains of proteoglycan core molecules. Chondroitinase has the potential to be a potent interfactant agent, given that this GAG is diffusely present at the vitreoretinal interface [228,238]. Despite a phase I/II FDA study demonstrating chondroitinase’s safety and some evidence of efficacy in inducing complete PVD without gel liquefaction, further trials were not conducted to test it for clinical use.

### 7.4. RGD Peptides

RGD peptides encompass the adhesive proteins present in ECMs which contain the tripeptide arginine-glycine-aspartic acid (RGD) at their cell recognition site. The specific binding complex RGD is important as it is present in various ECM components including laminin, fibronectin and certain collagens, thereby playing a potentially important role at the vitreoretinal interface. Because synthetic RGD peptides compete for integrin-binding sites, they could disrupt integrin–ECM interaction with subsequent loosening of interface attachments. Despite early encouraging studies in animal models [239], no further studies have tested the efficacy of RGD peptides on human eyes.

### 7.5. Dispase (Vitreolysin™)

Dispase cleaves the attachment of the posterior vitreous cortex to the ILM by degrading collagen IV and fibronectin. The low affinity of dispase for collagen II allows it to induce a PVD without disrupting the macromolecular structure of the vitreous body. However, dispase used at 0.025 U dosage or more caused injuries to the lens and retina in animal models, presumably due to its non-selective proteolytic activity that might involve degradation of non-vitreal proteic components [240].

### 7.6. Plasmin and Plasminogen Activators

Plasmin is a serine protease that weakens vitreoretinal adhesion attacking fibrin as well as other components of the ECM including fibronectin and laminin [241,242]. As it also moderately degrades the vitreous gel, plasmin can be considered either interfactant or liquefactant. Plasmin has a very good tolerability testified by the absence of side effects in preclinical studies [243,244,245]. One major drawback is its rapid inactivation and the lack of commercially available plasminogen, the proenzyme that must be activated soon before its use. To circumvent this problem, clinicians are required to purify plasminogen directly from patients’ plasma with a long and expensive procedure. Eventually, patients are injected with autologous plasmin enzyme (APE) [246]. APE has been employed in a variety of clinical situations [247,248,249]. However, because of the abovementioned drawbacks, APE cannot be routinely employed.

On the other hand, plasmin can be enzymatically generated in the vitreous cavity in the presence of plasminogen and plasminogen activators, such as tissue plasminogen activator (tPA) and urokinase. Spontaneous presence of plasminogen in the vitreal chamber, however, is observed only in pathological conditions which induce disruption of the blood–retinal barrier including PVR, PDR and hemorrhages [250]. Outside these specific clinical settings, plasminogen must be exogenously introduced [251], or its migration into the VH can be promoted by procedures (i.e., cryopexy) that provoke the disruption of the barrier [252]. Procedures such as the latter clearly pose problems of accurate dosage. All in all, the clinical use of plasminogen activators in ophthalmology is limited.

### 7.7. Ocriplasmin

Ocriplasmin is a truncated recombinant form of human plasmin. Formerly called microplasmin, this recombinant 27 kDa serine protease has a greater effect on thrombin compared to plasmin, and it binds to its primary inhibitor with weaker affinity. It exerts its proteolytic activity on laminin and fibronectin, both major components of the vitreous and of the vitreoretinal interface [253], and for this reason, it promotes simultaneous vitreous liquefaction and PVD. Ocriplasmin is the only vitreolytic agent approved for clinical use to treat selected cases of vitreoretinal interface disorders. Despite its significant superiority over a placebo in inducing the release of vitreo–macular adhesion and PVD [254], it should be noted that the non-specific enzymatic action of ocriplasmin may produce degradation of proteins in the vitreous, retina, choroid and lens, possibly causing several adverse ocular events [255].

### 7.8. Nattokinase

Nattokinase is a non-specific serine protease extracted from natto, a Japanese traditional food made from fermented soybean. Actually, it is produced by *Bacillus subtilis* which is found in natto. Nattokinase seems to promote the release of tPA from vascular endothelial cells, and this may explain the nattokinase ability to promote pharmacologic vitreolysis. The only study evaluating the effects of nattokinase demonstrated its efficacy to induce PVD in rabbits [256]. However, at the highest dose employed, nattokinase also induced preretinal hemorrhage and electroretinogram changes [256]. To our knowledge, there have been no further publications about nattokinase pharmacologic vitreolysis.

### 7.9. Vitreosolve^®^

Vitreosolve is a nonenzymatic agent for pharmacologic vitreolysis that contains urea. It is supposedly able to induce both liquefaction and vitreoretinal dehiscence. However, this molecule did not demonstrate superior efficacy in inducing PVD compared to a placebo in eyes with non-proliferative diabetic retinopathy, and clinical studies were discontinued [235].

### 7.10. Reenginered Vibrio mimicus Collagenase-Derived Fusion Proteins

Recent novel additions to the family of vitreolytic agents are a couple of reenginered forms of *Vibrio mimicus* collagenase-derived fusion proteins [257,258]. One is made of the collagen binding domain from the native *Vibrio mimicus* collagenase, characterized by the ability to destabilize the triple helix of collagen, fused with an RGD motif. Experimentally employed in rabbits, this agent was used in conjunction with the isolated collagen binding domain of the native collagenase determining PVD in 80% of cases. This outcome has been referred to the combined dehiscence properties of RGD peptides with the liquefaction ability of collagenase. Preliminary results show a good safety profile for this fusion protein [257]. The second reenginered form of *Vibrio mimicus* collagenase-derived fusion protein is made of a truncated and more stable form of the native *Vibrio mimicus* collagenase fused with a small peptide that is known to specifically bind to HA [258]. The latter modification is aimed to restrict the activity of the collagenase into the vitreous chamber. Preliminary results appear interesting either in terms of safety or in terms of effectiveness [258].

Both the above-discussed fusion proteins are in a very early stage of experimentation, and more data are needed to assess their clinical potentials.

## 8. Conclusions

Here, we have summarized the state of the art regarding our knowledge on the composition of the ECM of iERMs and its molecular arrangement, including the presence of deposits of LNPC matrix. Signs of an ordered organization appear particularly evident when considering the relationships occurring between collagen I and collagen IV. We have also highlighted how the lamellar pattern of ECM deposition can contribute to the tractional behavior of some iERMs. On the other hand, the ECM of iERMs is extremely complex. Knowledge of the intimate composition of the membranes and of the factors regulating its progress is essential to figure out the pathogenic mechanisms underlying their development. An interactome overview on proteins involved in ECM biology, previously identified with an MS-based proteomic analysis on iERM samples [13], unveils a set of proteins (i.e., ITGB1, EGFR, TGM2, LRP1, VEGF-A, galectin-3, CD44, cathepsins and cystatins, HTRA1, A2M) that, through a complex net of interactions with other iERM components, are likely central regulators of iERM pathogenesis and progression. The bioinformatic approach has provided a precious step forward in understanding the molecular bases of iERM development and in profiling novel pharmacologic targets for their treatment.

So far, drugs used to treat iERM are employed in surgical settings to promote vitreous and iERM detachment from the ILM. They are mainly derived from proteins with enzymatic activity purified from other contexts. Recently, fusion proteins specifically designed for targeting and acting on the vitreous to achieve vitreolysis have been synthesized and are the subjects of experiments. However, the interactome of iERM proteins extends far beyond the mere structural elements and offers a wider range of possible sites of pharmacological intervention. Even in this case, genetic engineering may offer the tool for synthesizing new drugs that might be employed to interfere with iERM development, its retracting capability and even to promote their regression.

## Data Availability

Not applicable.

## Supplementary Materials

The following supporting information can be downloaded at: https://www.mdpi.com/article/10.3390/cells11162531/s1, Figure S1: Shortest path net, trace mode visualization centred on VEGF-A. Only proteins that directly interact with VEGF-A (in bold) are visible. The rest of the network appears blurred in the background; Figure S2: Shortest path net, trace mode visualization centred on CD44. Only proteins that directly interact with CD44 (in bold) are visible. The rest of the network appears blurred in the background; Table S1: In order to facilitate net understanding, symbols used to represent the 141 experimental selected proteins in the generated nets are listed (column A) near the MetaCore protein names (column B). We also report the corresponding UniProtKB recommended protein names (column C) and identifiers (column D) of the corresponding UniProtKB entries. Column E provides the main compartmental localization of the listed proteins. Edge green-numbers (column F) indicate, for each row-protein, how many interactions are established by our list-proteins with other DIN hubs. Edge red-numbers (column G) specify, for each row-protein, the number of interactions selected iERM-proteins establish within the SPN.

## References

[1] Bertelli E., Bertelli E. (2019). Aqueous Humor, Lens, Ciliary Zonule, Vitreous. Anatomy of the Eye and Human Visual System.

[2] Tosi G.M., Marignani D., Romeo N., Toti P. (2014). Disease pathways in proliferative vitreoretinopathy: An ongoing challenge. J. Cell Physiol..

[3] Patronas M., Kroll A.J., Lou P.L., Ryan E.A. (2009). A review of the vitreoretinal interface pathology. Int. Ophthalmol. Clin..

[4] Folk J.C., Adelman R.A., Flaxel C.J., Hyman L., Pulido J.S., Olsen T.W. (2016). Idiopathic epiretinal membrane and vitreomacular traction preferred practice pattern (^®^) guidelines. Ophthalmology.

[5] Hiscott P.S., Grierson I., McLeod D. (1985). Natural history of fibrocellular epiretinal membranes: A quantitative, autoradiographic, and immunohistochemical study. Br. J. Ophthalmol..

[6] Sramek S.J., Wallow I.H., Stevens T.S., Nork T.M. (1989). Immunostaining of preretinal membranes for actin, fibronectin and glial fibrillary acidic protein. Ophthalmology.

[7] Bochaton-Piallat M.-L., Kapetanios A.D., Donati G., Redard M., Gabbiani G., Pournaras C.J. (2000). TGFβ1, TGFβ receptor II and ED-A fibronectin expression in myofibroblast of vitreoretinopathy. Investig. Ophthalmol. Vis. Sci..

[8] Cabay L., Willermain F., Bruyns C., Verderbout J.M., Witta Y., Baffi J., Velu T., Libert J., Caspers-Velu L., Maho A. (2003). CX3CR4 expression in vitreoretinal membranes. Br. J. Ophthalmol..

[9] Zhao F., Gandorfer A., Haritoglou C., Scheler R., Schaumberger M.M., Kampik A., Schumann R.G. (2013). Epiretinal cell proliferation in macular pucker and vitreomacular traction syndrome. Analysis of flat-mounted internal limiting membrane specimens. Retina.

[10] Schumann R.G., Gandorfer A., Ziada J., Scheler R., Schaumberger M.M., Wolf A., Kampik A., Haritoglou C. (2014). Hyalocytes in idiopathic epiretinal membranes: A correlative light and electron microscopic study. Graefes Arch. Clin. Exp. Ophthalmol..

[11] Bu S.-C., Kuijer R., van der Worp R.J., Postma G., de Lavalette R., Li X.-R., Hooymans J.M.M., Los L.I. (2015). Immunohistochemical evaluation of idiopathic epiretinal membranes and in vitro studies on the effect of TGFβ on Müller cells. Investig. Ophthalmol. Vis. Sci..

[12] Tosi G.M., Regoli M., Altera A., Galvagni F., Arcuri C., Bacci T., Elia I., Realini G., Orlandini M., Bertelli E. (2020). Heath shock protein 90 involvement in the development of idiopathic epiretinal membranes. Investig. Ophthalmol. Vis. Sci..

[13] Christakopoulos C., Cehofski L.J., Christensen S.R., Vorum H., Honore B. (2019). Proteomics reveals a set of highly enriched proteins in epiretina membrane compared with inner limiting membrane. Exp. Eye Res..

[14] Bini L., Schvartz D., Carnemolla C., Besio R., Garibaldi N., Sanchez J.C., Forlino A., Bianchi L. (2021). Intracellular and extracellular markers of lethality in osteogenesis imperfecta: A quantitative proteomic approach. Int. J. Mol. Sci..

[15] Bianchi L., Gagliardi A., Landi C., Focarelli R., De Leo V., Luddi A., Bini L., Piomboni P. (2016). Protein pathways working in human follicular fluid: The future for tailored IVF?. Expert Rev. Mol. Med..

[16] Bianchi L., Gagliardi A., Maruelli S., Besio R., Landi C., Gioia R., Kozloff K.M., Khoury B.M., Coucke P.J., Symoens S. (2015). Altered cytoskeletal organization characterized lethal but not surviving Brtl^+/−^ mice: Insight on phenotypic variability in osteogenesis imperfecta. Hum. Mol. Genet..

[17] Bianchi L., Bruzzese F., Leone A., Gagliardi A., Puglia M., Di Gennaro E., Rocco M., Gimigliano A., Pucci B., Armini A. (2011). Proteomic analysis identifies differentially expressed proteins after HDAC vorinostat and EGFR inhibitor gefitinib treatments in Hep-2 cancer cells. Proteomics.

[18] Okada M., Ogino N., Matsumura M., Honda Y., Nagai Y. (1995). Histological and immunohistochemical study of idiopathic epiretinal membrane. Ophthalmic Res..

[19] Kritzenberger M., Junglas B., Framme C., Helbig H., Gabel V.P., Fuchshofer R., Tamm E.R., Hillenkamp J. (2011). Different collagen types define two types of idiopathic epiretinal membranes. Histopathology.

[20] Regoli M., Tosi G.M., Neri G., Altera A., Orazioli D., Bertelli E. (2020). The peculiar pattern of type IV collagen deposition in epiretinal membranes. J. Histochem. Cytochem..

[21] Altera A., Tosi G.M., Regoli M., De Benedetto E., Bertelli E. (2021). The extracellular matrix complexity of idiopathic epiretinal membranes and the bilaminar arrangement of the associated internal limiting membrane in the posterior retina. Graefes Arch. Clin. Exp. Ophthalmol..

[22] Jerdan J.A., Pepose J.S., Michels R.G., Hayashi H., De Bustros S., Sebag M., Glaser B.M. (1989). Proliferative vitreoretinopathy membranes. An immunohistochemical study. Ophthalmology.

[23] Morino I., Hiscott P., McKechnie N., Grierson I. (1990). Variation in epiretinal membrane components with clinical duration of the proliferative tissue. Br. J. Ophthlamol..

[24] Guenther S.R., Schumann R.G., Hagenau F., Wolf A., Priglinger S.G., Vogt D. (2019). Comparison of surgically excised premacular membranes in eyes with macular pucker and proliferative vitreoretinopathy. Curr. Eye Res..

[25] George B., Chen S., Chaudhary V., Gonder J., Chakrabarti S. (2009). Extracellular matrix proteins in epiretinal membranes and in diabetic retinopathy. Curr. Eye Res..

[26] Coltrini D., Belleri M., Gambicorti E., Romano D., Morescalchi F., Chandran A.M.K., Calza S., Semeraro F., Presta M. (2020). Gene expression analysis identifies two distinct molecular clusters of idiopathic epiretinal membranes. Biochim. Biophys. Acta Mol. Basis Dis..

[27] Snead D.R.J., Cullen N., James S., Poulson A.V., Morris A.H.C., Lukaris A., Scott J.D., Richards A.J., Snead M.P. (2004). Hyperconvolution of the inner limiting membrane in vitreomaculopathies. Graefes Arch. Clin. Exp. Ophthamol..

[28] Asato R., Yoshida S., Ogura A., Nakam T., Ishikawa K., Nakao S., Sassa Y., Enaida H., Osima Y., Ikeo K. (2013). Comparison of gene expression profile of epiretinal membranes obtained from eyes with proliferative vitreoretinopathy to that of secondary epiretinal membranes. PLoS ONE.

[29] Fleischmajer R., Jacobs I.I.L., Perfish J.S., Katchen B., Schwartz E., Timpl R. (1992). Immunochemical analysis of human kidney reticulin. Am. J. Pathol..

[30] Yurchenko P.D. (2011). Basement membranes: Cell scaffoldings and signaling platforms. Cold Spring Harb. Perspect. Biol..

[31] Pratt B.M., Madri J.A. (1985). Immunolocalization of type IV collagen and laminin in nonbasement membrane structures of murine corneal stroma A light and electron microscopy. Lab. Investig..

[32] Oefner C.M., Sharkey A., Gardner L., Critchley H., Oyen M., Moffett A. (2015). Collagen type IV at the fetal-maternal interface. Placenta.

[33] Sharma A.K., Mauer S.M., Kim Y., Michael A.F. (1993). Interstitial fibrosis in obstructive nephropathy. Kidney Int..

[34] Urushiyama H., Terasaki Y., Nagasaka S., Terasaki M., Kunugi S., Nagase T., Fukuda Y., Shimizu A. (2015). Role of α1 and α2 chains of type IV collagen in early fibrotic lesions of idiopathic interstitial pneumonias and migration of lung fibroblasts. Lab. Investig..

[35] Ioachim E., Stefaniotou M., Gorezis S., Tsanou E., Psilas K., Agnantis N.J. (2005). Immunohistochemical study of extracellular matrix components in epiretinal membranes of vitreoproliferative retinopathy and proliferative diabetic retinopathy. Eur. J. Ophthalmol..

[36] Birk D.E. (2001). Type V collagen: Heterotypic type I/V collagen interactions in the regulation of fibril assembly. Micron.

[37] Bu S.C., Kuijer R., van der Worp R.J., Huiskamp E.A., Renardel de Lavalette V.W., Li X.R., Hooymans J.M., Los L.I. (2014). Glial cells and collagens in epiretinal membranes associated with idiopathic macular holes. Retina.

[38] Wullink B., Pas H.H., Ven der Worp R.J., Kuijer R., Los L.I. (2015). Type VII collagen expression in the human vitreoretinal interface, corpora amylacea and inner retinal layers. PLoS ONE.

[39] Ricard-Blum S. (2011). The collagen family. Cold Spring Harb. Perspect. Biol..

[40] Koch M., Schulze J., Hansen U., Ashwodt T., Keene D.R., Brunken W.J., Burgeson R.E., Bruckner P., Breucknes-Tuderman L. (2004). A novel marker of tissue junctions, collagen XXII. J. Biol. Chem..

[41] Halfter W., Dong S., Schurer B., Cole G.J. (1998). Collagen XVIII is a basement membrane heparin sulfate proteoglycan. J. Biol. Chem..

[42] Iozzo R.V., Schaefer L. (2015). Proteoglycan form and function: A comprehensive nomenclature of proteoglycans. Matrix Biol..

[43] Pastor-Pareja J.C. (2020). Atypical basement membranes and basement membrane diversity–what is normal anyway?. J. Cell Sci..

[44] Kvist A.J., Nyström A., Hultenby K., Sasaki T., Talts J.F., Aspberg A. (2008). The major basement membrane components localize to the chondrocyte pericellular matrix–a cartilage basement membrane equivalent?. Matrix Biol..

[45] Hagedorn M., Esser P., Wiedemann P., Heimann K. (1993). Tenascin and decorin in epiretinal membranes of proliferative vitreoretinopathy and proliferative diabetic retinopathy. Germ. J. Ophthalmol..

[46] Immonen I., Tervo K., Virtanen I., Laatikainen L., Tervo T. (1991). Immunohistochemical demonstration of cellular fibronectin and tenascin in human epiretinal membranes. Acta Ophthalmol..

[47] Pattwell D.M., Sheridan C.M., Le Goff M., Bishop P.N., Hiscott P. (2010). Localisation of opticin in human proliferative retinal disease. Exp. Eye Res..

[48] Alexander R.A., Hiscott P., McGalliard J., Grierson I. (1992). Oxytalan fibres in proliferative vitreoretinopathy. Germ. J. Ophthalmol..

[49] Weller M., Wiedemann P., Bresgen M., Heimann K. (1991). Vitronectin and proliferative intraocular disorders. I. A colocalisation study of the serum spreading factor, vitronectin and fibronectin in traction membranes from patients with proliferative vitreoretinopathy. Int. Ophthalmol..

[50] Hiscott P., Larkin G., Robey H.L., Orr G., Grierson I. (1992). Thrombospondin as a component of the extracellular matrix of epiretinal membranes: Comparisons with cellular fibronectin. Eye.

[51] Casaroli Marano R.P., Vilaró S. (1994). The role of fibronectin, laminin, vitronectin and their receptors on cellular adhesion in proliferative vitreoretinopathy. Investig. Ophthalmol. Vis. Sci..

[52] Grisanti S., Guidry C. (1995). Transdifferentiation of retinal pigment epithelial cells from epithelial to mesenchymal phenotype. Investig. Ophthalmol. Vis. Sci..

[53] Bornstein P. (2009). Matricellular proteins: An overview. J. Cell Commun. Signal..

[54] Weller M., Esser P., Bresgen M., Heimann K., Wiedemann P. (1992). Thrombospondin: A new attachment protein in preretinal traction membranes. Eur. J. Ophthalmol..

[55] Hiscott P., Hagan S., Heathcote L., Sheridan C.M., Gronewald C.P., Grierson I., Wong D., Paraoan L. (2002). Pathobiology of epiretinal and subretinal membranes: Possible roles for the matricellular proteins thrombospondin 1 and osteonectin (SPARC). Eye.

[56] Yoshida S., Nakama T., Oshokawa K., Kakao S., Sonoda K., Ishibashi T. (2017). Periostin in vitreoretinal diseases. Cell Mol. Life Sci..

[57] Russel S.R., Hageman G.S. (1992). Chondroitin sulfate-induced generation of epiretinal membranes. Arch. Ophthalmol..

[58] Azzolini C., Congiu T., Donati S., Passi A., Basso P., Piantanida E., Mariotti C., Testa F., Caprani S.M., Cattaneo J. (2017). Multilayer microstructure of idiopathic epiretinal macular membranes. Eur. J. Ophthalmol..

[59] Pollreisz A., Funk M., Breitwieser F.P., Parapatics K., Sacu S., Georgopoulos M., Dunavoelgyi R., Zlabinger G.J., Colinge J., Bennett K.L. (2013). Quantitative proteomics of aqueous and vitreous fluid from patients with idiopathic epiretinal membranes. Exp. Eye Res..

[60] Mandal N., Kofod M., Vorum H., Villumsen J., Erikeìsen J., Heegaard S., Prause J.U., Ahuja S., Nonoré B., la Cour M. (2013). Protemomic analysis of human vitreous associated with idiopathic epiretinal membranes. Acta Ophthalmol..

[61] Yu J., Feng L., Wu Y., Wang H., Ba J., Zhu W., Xie C. (2014). Vitreous proteomic analysis of idiopathic epiretinal membranes. Mol. Biosyst..

[62] Legate K.R., Wickström S.A., Fässler R. (2009). Genetic and cell biological analysis of integrin outside-in signaling. Genes Dev..

[63] Bachmann M., Kukkurainen S., Hytönen V.P., Wehrle-Haller B. (2019). Cell Adhesion by Integrins. Physiol. Rev..

[64] Torres-Gomez A., Cabañas C., Lafuente E.M. (2020). Phagocytic integrins: Activation and signaling. Front. Immunol..

[65] Cheng B., Wan W., Huang G., Li Y., Genin G.M., Mofrad M.R.K., Lu T.J., Xu F., Lin M. (2020). Nanoscale integrin cluster dynamics controls cellular mechanosensing via FAKY397 phosphorylation. Sci. Adv..

[66] Huang C., Ogawa R. (2012). Fibroproliferative disorders and their mechanobiology. Connect. Tissue Res..

[67] Leask A. (2013). Integrin *β*1: A mechanosignaling sensor essential for connective tissue deposition by fibroblasts. Adv. Wound Care.

[68] Reed N.I., Jo H., Chen C., Tsujino K., Arnold T.D., DeGrado W.F., Sheppard D. (2015). The αvβ1 integrin plays a critical in vivo role in tissue fibrosis. Sci. Transl. Med..

[69] Han Z., Ma Y., Cao G., Ma Z., Chen R., Cvijic M.E., Cheng D. (2021). Integrin αVβ1 regulates procollagen I production through a non-canonical transforming growth factor β signaling pathway in human hepatic stellate cells. Biochem. J..

[70] Boosani C.S., Mannam A.P., Cosgrove D., Silva R., Hodivala-Dilke K.M., Keshamouni V.G., Sudhakar A. (2007). Regulation of COX-2 mediated signaling by alpha3 type IV noncollagenous domain in tumor angiogenesis. Blood.

[71] Dedhar S., Jewell K., Rojiani M., Gray V. (1992). The receptor for the basement membrane glycoprotein entactin is the integrin alpha 3/beta 1. J. Biol. Chem..

[72] Kim K.K., Wei Y., Szekeres C., Kugler M.C., Wolters P.J., Hill M.L., Frank J.A., Brumwell A.N., Wheeler S.E., Kreidberg J.A. (2009). Epithelial cell alpha3beta1 integrin links beta-catenin and Smad signaling to promote myofibroblast formation and pulmonary fibrosis. J. Clin. Investig..

[73] Maldonado H., Hagood J.S. (2021). Cooperative signaling between integrins and growth factor receptors in fibrosis. J. Mol. Med..

[74] Wang X.Q., Sun P., Paller A.S. (2003). Ganglioside GM3 blocks the activation of epidermal growth factor receptor induced by integrin at specific tyrosine sites. J. Biol. Chem..

[75] Moro L., Dolce L., Cabodi S., Bergatto E., Boeri Erba E., Smeriglio M., Turco E., Retta S.F., Giuffrida M.G., Venturino M. (2002). Integrin-induced epidermal growth factor (EGF) receptor activation requires c-Src and p130Cas and leads to phosphorylation of specific EGF receptor tyrosines. J. Biol. Chem..

[76] Jeong B.Y., Cho K.H., Jeong K.J., Park Y.Y., Kim J.M., Rha S.Y., Park C.G., Mills G.B., Cheong J.H., Lee H.Y. (2018). Rab25 augments cancer cell invasiveness through a β1 integrin/EGFR/VEGF-A/Snail signaling axis and expression of fascin. Exp. Mol. Med..

[77] da Silva R.G., Tavora B., Robinson S.D., Reynolds L.E., Szekeres C., Lamar J., Batista S., Kostourou V., Germain M.A., Reynolds A.R. (2010). Endothelial alpha3beta1-integrin represses pathological angiogenesis and sustains endothelial-VEGF. Am. J. Pathol..

[78] Kanda A., Noda K., Hirose I., Ishida S. (2019). TGF-β-SNAIL axis induces Müller glial-mesenchymal transition in the pathogenesis of idiopathic epiretinal membrane. Sci. Rep..

[79] Hata Y., Sassa Y., Kita T., Miura M., Kano K., Kawahara S., Arita R., Nakao S., Shih J.L., Ishibashi T. (2008). Vascular endothelial growth factor expression by hyalocytes and its regulation by glucocorticoid. Br. J. Ophthalmol..

[80] Yafai Y., Iandiev I., Wiedemann P., Reichenbach A., Eichler W. (2004). Retinal endothelial angiogenic activity: Effects of hypoxia and glial (Müller) cells. Microcirculation.

[81] Smiddy W.E., Maguire A.M., Green W.R., Michels R.G., de la Cruz Z., Enger C., Jaeger M., Rice T.A. (1989). Idiopathic epiretinal membranes. Ultrastructural characteristics and clinicopathologic correlation. Ophthalmology.

[82] Eichler W., Yafai Y., Keller T., Wiedemann P., Reichenbach A. (2004). PEDF derived from glial Müller cells: A possible regulator of retinal angiogenesis. Exp. Cell Res..

[83] Araújo R.S., Santos D.F., Silva G.A. (2018). The role of the retinal pigment epithelium and Müller cells secretome in neovascular retinal pathologies. Biochimie.

[84] Bautch V.L. (2012). VEGF-directed blood vessel patterning: From cells to organism. Cold Spring Harb. Perspect. Med..

[85] Mac Gabhann F., Popel A.S. (2007). Dimerization of VEGF receptors and implications for signal transduction: A computational study. Biophys Chem..

[86] Cudmore M.J., Hewett P.W., Ahmad S., Wang K.Q., Cai M., Al-Ani B., Fujisawa T., Ma B., Sissaoui S., Ramma W. (2012). The role of heterodimerization between VEGFR-1 and VEGFR-2 in the regulation of endothelial cell homeostasis. Nat. Commun..

[87] Ma D.H., Yao J.Y., Kuo M.T., See L.C., Lin K.Y., Chen S.C., Chen J.K., Chao A.S., Wang S.F., Lin K.K. (2007). Generation of endostatin by matrix metalloproteinase and cathepsin from human limbocorneal epithelial cells cultivated on amniotic membrane. Investig. Ophthalmol. Vis. Sci..

[88] Walia A., Yang J.F., Huang Y.H., Rosenblatt M.I., Chang J.H., Azar D.T. (2015). Endostatin’s emerging roles in angiogenesis, lymphangiogenesis, disease, and clinical applications. Biochim. Biophys. Acta.

[89] Wee P., Wang Z. (2017). Epidermal growth factor receptor cell proliferation signaling pathways. Cancers.

[90] Shi Y., Liu N., Lai W., Yan B., Chen L., Liu S., Liu S., Wang X., Xiao D., Liu X. (2018). Nuclear EGFR-PKM2 axis induces cancer stem cell-like characteristics in irradiation-resistant cells. Cancer Lett..

[91] Epstein Shochet G., Brook E., Eyal O., Edelstein E., Shitrit D. (2019). Epidermal growth factor receptor paracrine upregulation in idiopathic pulmonary fibrosis fibroblasts is blocked by nintedanib. Am. J. Physiol. Lung Cell. Mol. Physiol..

[92] Panizo S., Martínez-Arias L., Alonso-Montes C., Cannata P., Martín-Carro B., Fernández-Martín J.L., Naves-Díaz M., Carrillo-López N., Cannata-Andía J.B. (2021). Fibrosis in chronic kidney disease: Pathogenesis and consequences. Int. J. Mol. Sci..

[93] Ren Y.X., Ma J.X., Zhao F., An J.B., Geng Y.X., Liu L.Y. (2018). Effects of curcumin on epidermal growth factor in proliferative vitreoretinopathy. Cell Physiol. Biochem..

[94] Li T., Zhang H.B., Meng J.M., Yuan B., Lin W.J., Feng Y., Chen X.D. (2021). YM155 inhibits retinal pigment epithelium cell survival through EGFR/MAPK signaling pathway. Int. J. Ophthalmol..

[95] Ni Y., Qin Y., Huang Z., Liu F., Zhang S., Zhang Z. (2020). Distinct serum and vitreous inflammation-related factor profiles in patients with proliferative vitreoretinopathy. Adv. Ther..

[96] Nabi I.R., Shankar J., Dennis J.W. (2015). The galectin lattice at a glance. J. Cell Sci..

[97] Cardoso A.C., Andrade L.N., Bustos S.O., Chammas R. (2016). Galectin-3 determines tumor cell adaptive strategies in stressed tumor microenvironments. Front. Oncol..

[98] Sedlář A., Trávníčková M., Bojarová P., Vlachová M., Slámová K., Křen V., Bačáková L. (2021). Interaction between galectin-3 and integrins mediates cell-matrix adhesion in endothelial cells and mesenchymal stem cells. Int. J. Mol. Sci..

[99] Slack R.J., Mills R., Mackinnon A.C. (2021). The therapeutic potential of galectin-3 inhibition in fibrotic disease. Int. J. Biochem. Cell Biol..

[100] Luis J., Eastlake K., Khaw P.T., Limb G.A. (2020). Galectins and their involvement in ocular disease and development. Exp. Eye Res..

[101] Mackinnon A.C., Gibbons M.A., Farnworth S.L., Leffler H., Nilsson U.J., Delaine T., Simpson A.J., Forbes S.J., Hirani N., Gauldie J. (2012). Regulation of transforming growth factor-β1-driven lung fibrosis by galectin-3. Am. J. Respir. Crit. Care Med..

[102] Henderson N.C., Mackinnon A.C., Farnworth S.L., Kipari T., Haslett C., Iredale J.P., Liu F.T., Hughes J., Sethi T. (2008). Galectin-3 expression and secretion links macrophages to the promotion of renal fibrosis. Am. J. Pathol..

[103] de Oliveira F.L., Panera N., De Stefanis C., Mosca A., D’Oria V., Crudele A., De Vito R., Nobili V., Alisi A. (2019). The number of liver galectin-3 positive cells is dually correlated with NAFLD severity in children. Int. J. Mol. Sci..

[104] Zhong X., Qian X., Chen G., Song X. (2019). The role of galectin-3 in heart failure and cardiovascular disease. Clin. Exp. Pharmacol. Physiol..

[105] Burguillos M.A., Svensson T., Schulte T., Boza-Serrano A., Garcia-Quintanilla A., Kavanagh E., Santiago M., Viceconte N., Oliva-Martin M.J., Osman A.M. (2015). Microglia-sereted galectin-3 acts as a toll-like receptor 4 ligand and contributes to microglial activation. Cell Rep..

[106] Díaz-Alvarez L., Ortega E. (2017). The many roles of galectin-3, a multifaceted molecule, in innate immune responses against pathogens. Mediators Inflamm..

[107] Woods E.L., Grigorieva I.V., Midgley A.C., Brown C.V.M., Lu Y.A., Phillips A.O., Bowen T., Meran S., Steadman R. (2021). CD147 mediates the CD44s-dependent differentiation of myofibroblasts driven by transforming growth factor-β_1_. J. Biol. Chem..

[108] Guindolet D., Gabison E.E. (2020). Role of CD147 (EMMPRIN/Basigin) in tissue remodeling. Anat. Rec..

[109] Arima M., Cui D., Kimura T., Sonoda K.H., Ishibashi T., Matsuda S., Ikeda E. (2016). Basigin can be a therapeutic target to restore the retinal vascular barrier function in the mouse model of diabetic retinopathy. Sci. Rep..

[110] Markowska A.I., Cao Z., Panjwani N. (2014). Glycobiology of ocular angiogenesis. Glycobiology.

[111] Patrizz A., Doran S.J., Chauhan A., Ahnstedt H., Roy-O’Reilly M., Lai Y.J., Weston G., Tarabishy S., Patel A.R., Verma R. (2020). EMMPRIN/CD147 plays a detrimental role in clinical and experimental ischemic stroke. Aging.

[112] Roesch K., Jadhav A.P., Trimarchi J.M., Stadler M.B., Roska B., Sun B.B., Cepko C.L. (2008). The transcriptome of retinal Müller glial cells. J. Comp. Neurol..

[113] Priglinger C.S., Szober C.M., Priglinger S.G., Merl J., Euler K.N., Kernt M., Gondi G., Behler J., Geerlof A., Kampik A. (2013). Galectin-3 induces clustering of CD147 and integrin-β1 transmembrane glycoprotein receptors on the RPE cell surface. PLoS ONE.

[114] Xu H., Manivannan A., Liversidge J., Sharp P.F., Forrester J.V., Crane I.J. (2002). Involvement of CD44 in leukocyte trafficking at the blood-retinal barrier. J. Leukoc. Biol..

[115] Sobue Y., Takahashi N., Ohashi Y., Suzuki M., Nishiume T., Kobayakawa T., Terabe K., Knudson W., Knudson C., Ishiguro N. (2019). Inhibition of CD44 intracellular domain production suppresses bovine articular chondrocyte de-differentiation induced by excessive mechanical stress loading. Sci. Rep..

[116] Chaitin M.H., Wortham H.S., Brun-Zinkernagel A.M. (1994). Immunocytochemical localization of CD44 in the mouse retina. Exp. Eye Res..

[117] Nishina S., Hirakata A., Hida T., Sawa H., Azuma N. (1997). CD44 expression in the developing human retina. Graefes Arch. Clin. Exp. Ophthalmol..

[118] Yan X., Andresen P., Lumi X., Chen Q., Petrovski G. (2018). Expression of progenitor cell markers in the glial-like cells of epiretinal membranes of different origins. J. Ophthalmol..

[119] Abu El-Asrar A.M., Missotten L., Geboes K. (2011). Expression of myofibroblast activation molecules in proliferve vitreoretinopathy epiretinal membranes. Acta Ophthalmol..

[120] Kampik A., Kenyon K.R., Michels R.G., Green W.R., de la Cruz Z.C. (1981). Epiretinal and vitreous membranes. Comparative study of 56 cases. Arch. Ophthalmol..

[121] McKeown-Longo P.J., Higgins P.J. (2020). Hyaluronan, transforming growth factor β, and extra domain A-fibronectin: A fibrotic triad. Adv. Wound Care.

[122] Ko M.K., Saraswathy S., Parikh J.G., Rao N.A. (2011). The role of TLR4 activation in photoreceptor mitochondrial oxidative stress. Investig. Ophthalmol. Vis. Sci..

[123] Jiang G., Sun D., Kaplan H.J., Shao H. (2012). Retinal astrocytes pretreated with NOD2 and TLR2 ligands activate uveitogenic T cells. PLoS ONE.

[124] Midgley A.C., Rogers M., Hallett M.B., Clayton A., Bowen T., Phillips A.O., Steadman R. (2013). Transforming growth factor-β1 (TGF-β1)-stimulated fibroblast to myofibroblast differentiation is mediated by hyaluronan (HA)-facilitated epidermal growth factor receptor (EGFR) and CD44 co-localization in lipid rafts. J. Biol. Chem..

[125] Shi X., Leng L., Wang T., Wang W., Du X., Li J., McDonald C., Chen Z., Murphy J.W., Lolis E. (2006). CD44 is the signaling component of the macrophage migration inhibitory factor-CD74 receptor complex. Immunity.

[126] Osipyan A., Chen D., Dekker F.J. (2021). Epigenetic regulation in macrophage migration inhibitory factor (MIF)-mediated signaling in cancer and inflammation. Drug Discov. Today.

[127] Kleemann R., Hausser A., Geiger G., Mischke R., Burger-Kentischer A., Flieger O., Johannes F.J., Roger T., Calandra T., Kapurniotu A. (2000). Intracellular action of the cytokine MIF to modulate AP-1 activity and the cell cycle through Jab1. Nature.

[128] Man A.L., Lodi F., Bertelli E., Regoli M., Pin C., Mulholland F., Satoskar A.R., Taussig M.J., Nicoletti C. (2008). Macrophage migration inhibitory factor plays a role in the regulation of microfold (M) cell-mediated transport in the gut. J. Immunol..

[129] Abu El-Asrar A.M., Ahmad A., Siddiquei M.M., De Zutter A., Allegaert E., Gikandi P.W., De Hertogh G., Van Damme J., Opdenakker G., Struyf S. (2019). The proinflammatory and proangiogenic macrophage migration inhibitory factor is a potential regulator in proliferative diabetic retinopathy. Front. Immunol..

[130] Turk B., Turk D., Turk V. (2012). Protease signalling: The cutting edge. EMBO J.

[131] Garcia-Cattaneo A., Gobert F.X., Müller M., Toscano F., Flores M., Lescure A., Del Nery E., Benaroch P. (2012). Cleavage of Toll-like receptor 3 by cathepsins B and H is essential for signaling. Proc. Natl. Acad. Sci. USA.

[132] Chevriaux A., Pilot T., Derangère V., Simonin H., Martine P., Chalmin F., Ghiringhelli F., Rébé C. (2020). Cathepsin B is required for NLRP3 inflammasome activation in macrophages, through NLRP3 interaction. Front. Cell Dev. Biol..

[133] Unanue E.R., Turk V., Neefjes J. (2016). Variations in MHC Class II Antigen Processing and Presentation in Health and Disease. Annu. Rev. Immunol..

[134] Vishwakarma S., Gupta R.K., Jakati S., Tyagi M., Pappuru R.R., Reddig K., Hendricks G., Volkert M.R., Khanna H., Chhablani J. (2020). Molecular assessment of epiretinal membrane: Activated microglia, oxidative stress and inflammation. Antioxidants.

[135] Joshi M., Agrawal S., Christoforidis J.B. (2013). Inflammatory mechanisms of idiopathic epiretinal membrane formation. Mediators Inflamm..

[136] Yadati T., Houben T., Bitorina A., Shiri-Sverdlov R. (2020). *The ins and outs of cathepsins*: Physiological function and role in disease management. Cells.

[137] Buck M.R., Karustis D.G., Day N.A., Honn K.V., Sloane B.F. (1992). Degradation of extracellular-matrix proteins by human cathepsin B from normal and tumour tissues. Biochem. J..

[138] Christensen J., Shastri V.P. (2015). Matrix-metalloproteinase-9 is cleaved and activated by cathepsin K. BMC. Res. Notes.

[139] Ruan H., Hao S., Young P., Zhang H. (2015). Targeting cathepsin B for cancer therapies. Horiz. Cancer Res..

[140] Ricard-Blum S., Vallet S. (2019). Fragments generated upon extracellular matrix remodelling: Biological regulators and potential drugs. Matrix Biol..

[141] Wells J.M., Gaggar A., Blalock J.E. (2015). MMP generated matrikines. Matrix Biol..

[142] Szmola R., Kukor Z., Sahin-Tóth M. (2003). Human mesotrypsin is a unique digestive protease specialized for the degradation of trypsin inhibitors. J. Biol. Chem..

[143] Paraoan L., Sharif U., Carlsson E., Supharattanasitthi W., Mahmud N.M., Kamalden T.A., Hiscott P., Jackson M., Grierson I. (2020). Secretory proteostasis of the retinal pigmented epithelium: Impairment links to age-related macular degeneration. Prog. Retin. Eye Res..

[144] Tinklepaugh J., Smith B.M., Nie Y., Moody K., Grohn K., Bou-Abdallah F., Doyle R.P. (2017). Saposin B binds the lipofuscin bisretinoid A2E and prevents its enzymatic and photooxidation. ChemPhotoChem.

[145] Morrone C., Smirnova N.F., Jeridi A., Kneidinger N., Hollauer C., Schupp J.C., Kaminski N., Jenne D., Eickelberg O., Yildirim A.Ö. (2021). Cathepsin B promotes collagen biosynthesis, which drives bronchiolitis obliterans syndrome. Eur. Respir. J..

[146] Kasabova M., Joulin-Giet A., Lecaille F., Gilmore B.F., Marchand-Adam S., Saidi A., Lalmanach G. (2014). Regulation of TGF-β1-driven differentiation of human lung fibroblasts: Emerging roles of cathepsin B and cystatin C. J. Biol. Chem..

[147] Moles A., Tarrats N., Fernández-Checa J.C., Marí M. (2009). Cathepsins B and D drive hepatic stellate cell proliferation and promote their fibrogenic potential. Hepatology.

[148] Priglinger S.G., May C.A., Neubauer A.S., Alge C.S., Schönfeld C.L., Kampik A., Welge-Lussen U. (2003). Tissue transglutaminase as a modifying enzyme of the extracellular matrix in PVR membranes. Investig. Ophthalmol. Vis. Sci..

[149] Quan G., Choi J.Y., Lee D.S., Lee S.C. (2005). TGF-beta1 up-regulates transglutaminase two and fibronectin in dermal fibroblasts: A possible mechanism for the stabilization of tissue inflammation. Arch. Dermatol. Res..

[150] Akimov S.S., Belkin A.M. (2001). Cell-surface transglutaminase promotes fibronectin assembly via interaction with the gelatin-binding domain of fibronectin: A role in TGFbeta-dependent matrix deposition. J. Cell Sci..

[151] Tovar-Vidales T., Clark A.F., Wordinger R.J. (2011). Focus on molecules: Transglutaminase 2. Exp. Eye Res..

[152] Aeschlimann D., Paulsson M., Mann K. (1992). Identification of Gln726 in nidogen as the amine acceptor in transglutaminase-catalyzed crosslinking of laminin–nidogen complexes. J. Biol. Chem..

[153] Kleman J.P., Aeschlimann D., Paulsson M., van der Rest M. (1995). Transglutaminase-catalyzed cross-linking of fibrils of collagen V⁄XI in A204 rhabdomyosarcoma cells. Biochemistry.

[154] Lai T.S., Lin C.J., Greenberg C.S. (2017). Role of tissue transglutaminase-2 (TG2)-mediated aminylation in biological processes. Amino Acids.

[155] Melkonian A.V., Weng N., Palanski B.A., Khosla C. (2019). In vivo measurement of redox-regulated TG2 activity. Methods Mol. Biol..

[156] Lai T.S., Lin C.J., Wu Y.T., Wu C.J. (2017). Tissue transglutaminase (TG2) and mitochondrial function and dysfunction. Front. Biosci..

[157] Campisi A., Caccamo D., Li Volti G., Currò M., Parisi G., Avola R., Vanella A., Ientile R. (2004). Glutamate-evoked redox state alterations are involved in tissue transglutaminase upregulation in primary astrocyte cultures. FEBS Lett..

[158] Kim Y., Eom S., Kim K., Lee Y.S., Choe J., Hahn J.H., Lee H., Kim Y.M., Ha K.S., Ro J.Y. (2010). Transglutaminase II interacts with rac1, regulates production of reactive oxygen species, expression of snail, secretion of Th2 cytokines and mediates in vitro and in vivo allergic inflammation. Mol. Immunol..

[159] Barrallo-Gimeno A., Nieto M.A. (2005). The Snail genes as inducers of cell movement and survival: Implications in development and cancer. Development.

[160] Li H., Wang H., Wang F., Gu Q., Xu X. (2011). Snail involves in the transforming growth factor β1-mediated epithelial-mesenchymal transition of retinal pigment epithelial cells. PLoS ONE.

[161] Oka C., Tsujimoto R., Kajikawa M., Koshiba-Takeuchi K., Ina J., Yano M., Tsuchiya A., Ueta Y., Soma A., Kanda H. (2004). HtrA1 serine protease inhibits signaling mediated by Tgfbeta family proteins. Development.

[162] Yamawaki S., Naitoh M., Kubota H., Aya R., Katayama Y., Ishiko T., Tamura T., Yoshikawa K., Enoshiri T., Ikeda M. (2018). HtrA1 is specifically up-regulated in active keloid lesions and stimulates keloid development. Int. J. Mol. Sci..

[163] Owen L.A., Shirer K., Collazo S.A., Szczotka K., Baker S., Wood B., Carroll L., Haaland B., Iwata T., Katikaneni L.D. (2020). The serine protease HTRA-1 is a biomarker for ROP and mediates retinal neovascularization. Front. Mol. Neurosci..

[164] Yu T., Chen C.Z., Xing Y.Q. (2017). Inhibition of cell proliferation, migration and apoptosis in blue-light illuminated human retinal pigment epithelium cells by down-regulation of HtrA1. Int. J. Ophthalmol..

[165] Acosta H., Iliev D., Grahn T.H., Gouignard N., Maccarana M., Griesbach J., Herzmann S., Sagha M., Climent M., Pera E.M. (2015). The serpin PN1 is a feedback regulator of FGF signaling in germ layer and primary axis formation. Development.

[166] Romay M.C., Toro C., Iruela-Arispe M.L. (2019). Emerging molecular mechanisms of vascular dementia. Curr. Opin. Hematol..

[167] Romaniuk D., Kimsa M.W., Strzalka-Mrozik B., Kimsa M.C., Kabiesz A., Romaniuk W., Mazurek U. (2013). Gene expression of IGF1, IGF1R, and IGFBP3 in epiretinal membranes of patients with proliferative diabetic retinopathy: Preliminary study. Mediators Inflamm..

[168] Harada T., Harada C., Mitamura Y., Akazawa C., Ohtsuka K., Ohno S., Takeuchi S., Wada K. (2002). Neurotrophic factor receptors in epiretinal membranes after human diabetic retinopathy. Diabetes Care.

[169] Kuznetsova A.V., Rzhanovaa L.A., Kurinova A.M., Aleksandrova M.A. (2019). The effect of basic fibroblast growth factor on signaling pathways in adult human retinal pigment epithelial cells. Cell Tissue Biol..

[170] Rehman A.A., Ahsan H., Khan F.H. (2013). α-2-Macroglobulin: A physiological guardian. J. Cell Physiol..

[171] Bres E.E., Faissner A. (2019). Low density receptor-related protein 1 interactions with the extracellular matrix: More than meets the eye. Front. Cell Dev. Biol..

[172] Schnieder J., Mamazhakypov A., Birnhuber A., Wilhelm J., Kwapiszewska G., Ruppert C., Markart P., Wujak L., Rubio K., Barreto G. (2020). Loss of LRP1 promotes acquisition of contractile-myofibroblast phenotype and release of active TGF-β1 from ECM stores. Matrix Biol..

[173] Rabiej V.K., Pflanzner T., Wagner T., Goetze K., Storck S.E., Eble J.A., Weggen S., Mueller-Klieser W., Pietrzik C.U. (2016). Low density lipoprotein receptor-related protein 1 mediated endocytosis of β1-integrin influences cell adhesion and cell migration. Exp. Cell Res..

[174] Strickland D.K., Muratoglu S.C., Antalis T.M. (2011). Serpin-enzyme receptors LDL receptor-related protein 1. Methods Enzymol..

[175] Salicioni A.M., Gaultier A., Brownlee C., Cheezum M.K., Gonias S.L. (2004). Low density lipoprotein receptor-related protein-1 promotes beta1 integrin maturation and transport to the cell surface. J. Biol. Chem..

[176] Wujak L., Böttcher R.T., Pak O., Frey H., El Agha E., Chen Y., Schmitt S., Bellusci S., Schaefer L., Weissmann N. (2018). Low density lipoprotein receptor-related protein 1 couples β1 integrin activation to degradation. Cell Mol. Life Sci..

[177] Zemskov E.A., Mikhailenko I., Strickland D.K., Belkin A.M. (2007). Cell-surface transglutaminase undergoes internalization and lysosomal degradation: An essential role for LRP1. J. Cell Sci..

[178] Jaeschke A., Hui D.Y. (2021). LDL receptor-related protein 1 and its interacting partners in tissue homeostasis. Curr. Opin. Lipidol..

[179] Wujak L., Schnieder J., Schaefer L., Wygrecka M. (2018). LRP1: A chameleon receptor of lung inflammation and repair. Matrix Biol..

[180] Barcelona P.F., Jaldín-Fincati J.R., Sánchez M.C., Chiabrando G.A. (2013). Activated α2-macroglobulin induces Müller glial cell migration by regulating MT1-MMP activity through LRP1. FASEB J..

[181] Mansour A.M., Chess J., Henkind P. (1987). Fibrinogen-induced vitreous membranes. Ophthalmic. Res..

[182] Zhang P., Zhu M., Zhao Y., Qian J., Dufresne C., Turner R., Semba R.D., Solomon S.D. (2017). A proteomic approach to understanding the pathogenesis of idiopathic macular hole formation. Clin. Proteom..

[183] Trink J., Li R., Palarasah Y., Troyanov S., Andersen T.E., Sidelmann J.J., Inman M.D., Pizzo S.V., Gao B., Krepinsky J.C. (2021). Activated alpha 2-macroglobulin is a novel mediator of mesangial cell profibrotic signaling in diabetic kidney disease. Biomedicines.

[184] Khalkhali-Ellis Z., Hendrix M.J. (2007). Elucidating the function of secreted maspin: Inhibiting cathepsin D-mediated matrix degradation. Cancer Res..

[185] Kurz S., Thieme R., Amberg R., Groth M., Jahnke H.G., Pieroh P., Horn L.C., Kolb M., Huse K., Platzer M. (2017). The anti-tumorigenic activity of A2M-A lesson from the naked mole-rat. PLoS ONE.

[186] Tosi G.M., Neri G., Caldi E., Fusco F., Bacci T., Tarantello A., Nuti E., Marigliani D., Baiocchi S., Traversi C. (2018). TGF-β concentrations and activity are down-regulated in the aqueous humor of patients with neovascular age-related macular degeneration. Sci. Rep..

[187] Tosi G.M., Orlandini M., Galvagni F. (2018). The controversial role of TGF-β in neovascular age-related macular degeneration pathogenesis. Int. J. Mol. Sci..

[188] Hashimoto R., Jiang M., Shiba T., Hiruta N., Takahashi M., Higashi M., Hori Y., Bujo H., Maeno T. (2017). Soluble form of LR11 is highly increased in the vitreous fluids of patients with idiopathic epiretinal membrane. Graefes Arch. Clin. Exp. Ophthalmol..

[189] Iannetti L., Accorinti M., Malagola R., Bozzoni-Pantaleoni F., Da Dalt S., Nicoletti F., Gradini R., Traficante A., Campanella M., Pivetti-Pezzi P. (2011). Role of the intravitreal growth factors in the pathogenesis of idiopathic epiretinal membrane. Investig. Ophthalmol. Vis. Sci..

[190] Jiang M., Bujo H., Ohwaki K., Unoki H., Yamazaki H., Kanaki T., Shibasaki M., Azuma K., Harigaya K., Schneider W.J. (2008). Ang II-stimulated migration of vascular smooth muscle cells is dependent on LR11 in mice. J. Clin. Investig..

[191] Shiba T., Bujo H., Takahashi M., Sato Y., Jiang M., Hori Y., Maeno T., Shirai K. (2013). Vitreous fluid and circulating levels of soluble lr11, a novel marker for progression of diabetic retinopathy. Graefes Arch. Clin. Exp. Ophthalmol..

[192] Yao H., Ge T., Zhang Y., Li M., Yang S., Li H., Wang F. (2019). BMP7 antagonizes proliferative vitreoretinopathy through retinal pigment epithelial fibrosis in vivo and in vitro. FASEB J..

[193] Zandi S., Tappeiner C., Pfister I.B., Despont A., Rieben R., Garweg J.G. (2016). Vitreal cytokine profile differences between eyes with epiretinal membranes or macular holes. Investig. Ophthalmol. Vis. Sci..

[194] Minchiotti S., Stampachiacchiere B., Micera A., Lambiase A., Ripandelli G., Billi B., Bonini S. (2008). Human idiopathic epiretinal membranes express NGF and NGF receptors. Retina..

[195] Chen Y.S., Hackett S.F., Schoenfeld C.L., Vinores M.A., Vinores S.A., Campochiaro P.A. (1997). Localisation of vascular endothelial growth factor and its receptors to cells of vascular and avascular epiretinal membranes. Br. J. Ophthalmol..

[196] Simm A., Nestler M., Hoppe V. (1997). PDGF-AA, a potent mitogen for cardiac fibroblasts from adult rats. J. Mol. Cell. Cardiol..

[197] Juhl P., Bondesen S., Hawkins C.L., Lambiase A., Ripandelli G., Billi B., Siebuhr A.S. (2020). Dermal fibroblasts have different extracellular matrix profiles induced by TGF-β, PDGF and IL-6 in a model for skin fibrosis. Sci. Rep..

[198] Robbins S.G., Mixon R.N., Wilson D.J., Hart C.E., Robertson J.E., Westra I., Planck S.R., Rosenbaum J.T. (1994). Platelet-derived growth factor ligands and receptors immunolocalized in proliferative retinal disease. Investig. Ophthalmol. Vis. Sci..

[199] Cui J.Z., Chiu A., Maberley D., Ma P., Samad A., Matsubara J.A. (2007). Stage specificity of novel growth factor expression during development of proliferative vitreoretinopathy. Eye.

[200] Micera A., Vigneti E., Pickholtz D., Ma P., Samad A., Matsubara J.A., Maquart F.X., Aloe L., Levi-Schaffer F. (2001). Nerve growth factor displays stimulatory effects on human skin and lung fibroblasts, demonstrating a direct role for this factor in tissue repair. Proc Natl Acad Sci, USA.

[201] Liu Z., Cao Y., Liu G., Yin S., Ma J., Liu J., Zhang M., Wang Y. (2019). p75 neurotrophin receptor regulates NGF-induced myofibroblast differentiation and collagen synthesis through MRTF-A. Exp. Cell Res..

[202] Kılıç A., Sonar S.S., Yildirim A.O., Fehrenbach H., Nockher W.A., Renz H. (2011). Nerve growth factor induces type III collagen production in chronic allergic airway inflammation. J. Allergy Clin. Immunol..

[203] Chung C.W., Zhang Q.L., Qiao L.-Y. (2010). Endogenous nerve growth factor regulates collagen expression and bladder hypertrophy through Akt and MAPK pathways during cystitis. J. Biol. Chem..

[204] Armstrong D., Augustin A.J., Spengler R., Al-Jada A., Nickola T., Grus F., Koch F. (1998). Detection of vascular endothelial growth factor and tumor necrosis factor alpha in epiretinal membranes of proliferative diabetic retinopathy, proliferative vitreoretinopathy and macular pucker. Ophthalmologica.

[205] Watanabe D., Suzuma K., Suzuma I., Ohashi H., Ojima T., Kurimoto M., Murakami T., Kimura T., Takagi H. (2005). Vitreous levels of angiopoietin 2 and vascular endothelial growth factor in patients with proliferative diabetic retinopathy. Am. J. Ophthalmol..

[206] Applewhite B.P., Babapoor-Farrokhran S., Poon D., Hassan S.J., Wellmann E., Ying H.S., Semenza G.L., Montaner S., Sodhi A. (2017). Lack of evidence for vasoactive and inflammatory mediators in the promotion of macular edema associated with epiretinal membranes. Sci. Rep..

[207] Harada C., Mitamura Y., Harada T. (2006). The role of cytokines and trophic factors in epiretinal membranes: Involvement of signal transduction in glial cells. Prog. Retin. Eye Res..

[208] Tosi G.M., Caldi E., Neri G., Nuti E., Marigliani D., Baiocchi S., Traversi C., Cevenini G., Tarantello A., Fusco F. (2017). HTRA1 and TGF-β1 concentrations in the aqueous humor of patients with neovascular age-related macular degeneration. Investig. Ophthalmol. Vis. Sci..

[209] Saint-Geniez M., Maharaj A.S., Walshe T.E., Tucker B.A., Sekiyama E., Kurihara T., Darland D.C., Young M.J., D’Amore P.A. (2008). Endogenous VEGF is required for visual function: Evidence for a survival role on müller cells and photoreceptors. PLoS ONE.

[210] Myojin S., Yoshimura T., Yoshida S., Takeda A., Murakami Y., Kawano Y., Oshima Y., Ishibashi T., Sonoda K.H. (2016). Gene expression analysis of the irrigation solution samples collected during vitrectomy for idiopathic epiretinal membrane. PLoS ONE.

[211] Russo A., Ragusa M., Barbagallo C., Longo A., Avitabile T., Uva M.G., Bonfiglio V., Toro M.D., Caltabiano R., Mariotti C. (2017). miRNAs in the vitreous humor of patients affected by idiopathic epiretinal membrane and macular hole. PLoS ONE.

[212] Mestdagh P., Boström A.K., Impens F., Fredlund E., Van Peer G., De Antonellis P., von Stedingk K., Ghesquière B., Schulte S., Dews M. (2010). The miR-17-92 microRNA cluster regulates multiple components of the TGF-β pathway in neuroblastoma. Mol. Cell..

[213] Dews M., Fox J.L., Hultine S., Sundaram P., Wang W., Liu Y.Y., Furth E., Enders G.H., El-Deiry W., Schelter J.M. (2010). The myc-miR-17~92 axis blunts TGF{beta} signaling and production of multiple TGF{beta}-dependent antiangiogenic factors. Cancer Res.

[214] Sun Q., Zhang Y., Yang G., Chen X., Zhang Y., Cao G., Wang J., Sun Y., Zhang P., Fan M. (2008). Transforming growth factor-beta-regulated miR-24 promotes skeletal muscle differentiation. Nucleic Acids Res..

[215] Chan M.C., Hilyard A.C., Wu C., Davis B.N., Hill N.S., Lal A., Lieberman J., Lagna G., Hata A. (2010). Molecular basis for antagonism between PDGF and the TGFbeta family of signalling pathways by control of miR-24 expression. EMBO J..

[216] Wickham L., Gregor Z., Ryan S.J. (2013). Epiretinal Membranes. Retina.

[217] Yusuf A.M., Bizrah M., Bunce C., Bainbridge J.W. (2021). Surgery for idiopathic epiretinal membrane. Cochrane Database Syst. Rev..

[218] Stein J.D., Zacks D.N., Grossman D., Grabe H., Johnson M.W., Sloan F.A. (2009). Trends in rates of adverse events after pars plana vitrectomy among medicare beneficiaries. Arch. Ophthalmol..

[219] Sebag J. (1998). Pharmacologic vitreolysis. Retina.

[220] de Smet M.D., Gandorfer A., Stalmans P., Veckeneer M., Feron E., Pakola S., Kampik A. (2009). Microplasmin intravitreal administration in patients with vitreomacular traction scheduled for vitrectomy. Ophthalmology.

[221] Stalmans P., Delaey C., de Smet M.D., van Dijkman E., Pakola S. (2010). Intravitreal injection of microplasmin for treatment of vitreomacular adhesion: Results of a prospective, randomized, sham-controlled phase II trial (the MIVI-IIT trial). Retina.

[222] Stalmans P., Benz M.S., Gandorfer A., van Dijkman E., Pakola S. (2012). Enzymatic vitreolysis with ocriplasmin for vitreomacular traction and macular holes. N. Engl. J. Med..

[223] Kampik A. (2012). Pathology of epiretinal membrane, idiopathic macular hole, and vitreomacular traction syndrome. Retina.

[224] Trese M.T. (2002). Enzymatic-assisted vitrectomy. Eye.

[225] Schumann R.G., Wolf A., Mayer W.J., Compera D., Hagenau F., Ziada J., Kampik A., Haritoglou C. (2015). Pathology of internal limiting membrane specimens following intravitreal injection of ocriplasmin. Am. J. Ophthalmol..

[226] Kohno T., Sorgenre N., Ishibashi T., Goodnight R., Ryan S.J. (1987). Immunofluorescent studies of fibronectin and laminin in the humon eye. Investig. Ophthalmol. Vis. Sci..

[227] Ponsioen T.L., van Luyn M.J.A., van der Worp R.J., van Meurs J.C., Hooymans J.M., Los L.I. (2008). Collagen distribution in the human vitreoretinal interface. Investig. Ophthalmol. Vis. Sci..

[228] Clark S.J., Keenan T.D.L., Fielder H.L., Collinson L.J., Holley R.J., Merry C.L.R., Kampik A., Haritoglou C. (2011). Mapping the differential distribution of glycosaminoglycans in the adult human retina, choroid, and sclera. Investig. Ophthalmol. Vis. Sci..

[229] Keenan T.D.L., Clark S.J., Unwin R.D., Ridge L.A., Day A.J., Bishop P.N. (2012). Mapping the differential distribution of proteoglycan core proteins in the adult human retina, choroid, and sclera. Investig. Ophthalmol. Vis. Sci..

[230] Halfter W., Monnier C., Müller D., Oertle P., Uechi G., Balasubramani M., Safi F., Lim R., Loparic M., Henrich P.B. (2013). The bi-functional organization of human basement membranes. PLoS ONE.

[231] Schneider E.W., Johnson M.W. (2011). Emerging non surgical methods for the treatment of vitreomacular adhesion: A review. Clin. Ophthalmol..

[232] Koleva-Georgieva D.N. (2015). Pharmacologic vitreolysis: New strategy for treatment of anomalous vitreo-macular adhesion. World. J. Ophthalmol..

[233] Dolz-Marco R., Gallego-Pinazo R., Diaz-Llopis M., Arévalo J.F. (2014). Pharmacovitrectomy. Dev. Ophthalmol..

[234] Wan R., Hong T., Tariq Y., Chang A. (2018). Pharmacotherapy of vitreomacular traction. Curr. Pharm. Des..

[235] Sebag J., Sebag J. (2014). Pharmacologic Vitreolysis. Vitreous in Health and Disease.

[236] Moorhead L.C., Redburn D.A., Kirkpatrick D.S., Kretzer F. (1980). Bacterial collagenase. Proposed adjunct to vitrectomy with membranectomy. Arch. Ophthalmol..

[237] Kuppermann B.D., Thomas E.L., de Smet M.D., Grillone L.R. (2005). Pooled efficacy results from two multinational randomized controlled clinical trials of a single intravitreous injection of highly purified ovine hyaluronidase (Vitrase^®^) for the management of vitreous hemorrhage. Am. J. Ophthalmol..

[238] Russell S.R. (2012). What we know (and what do not know) about vitreoretinal adhesion. Retina..

[239] Oliveira L.B., Meyer C.H., Kumar J., Tatebayashi M., Toth C.A., Wong F., Epstein D.L., McCuen B.W. (2002). RGD peptide-assisted vitrectomy to facilitate induction of a posterior vitreous detachment: A new principle in pharmacological vitreolysis. Curr. Eye Res..

[240] Wang F., Wang Z., Sun X., Wang F., Xu X., Zhang X. (2004). Safety and efficacy of dispase and plasmin in pharmacologic vitreolysis. Investig. Ophthalmol. Vis. Sci..

[241] Liotta L.A., Goldfarb R.H., Brundage R., Siegal G.P., Terranova V., Garbisa S. (1981). Effect of plasminogen activator (urokinase), plasmin, and thrombin on glycoprotein and collagenous components of basement membrane. Cancer Res..

[242] Uemura A., Nakamura M., Kachi S., Nishizawa Y., Asami T., Miyake Y., Terasaki H. (2005). Effect of plasmin on laminin and fibronectin during plasmin-assisted vitrectomy. Arch. Ophthalmol..

[243] Hikichi T., Yanagiya N., Kado M., Akiba J., Yoshida A. (1999). Posterior vitreous detachment induced by injection of plasmin and sulfur hexafluoride in the rabbit vitreous. Retina.

[244] Gandorfer A., Putz E., Welge-Lussen U., Gruterich M., Ulbig M., Kampik A. (2001). Ultrastructure of the vitreoretinal interface following plasmin assisted vitrectomy. Br. J. Ophthalmol..

[245] Gandorfer A., Priglinger S., Schebitz K., Hoops J., Ulbig M., Ruckhofer J., Grabner G., Kampik A. (2002). Vitreoretinal morphology of plasmin treated human eyes. Am. J. Ophthalmol..

[246] Rizzo S., Pellegrini G., Benocci F., Belting C., Baicchi U., Vispi M. (2006). Autologous plasmin for pharmacologic vitreolysis prepared 1 hour before surgery. Retina.

[247] Tsukahara Y., Honda S., Imai H., Kondo N., Fujii S., Yokoyama N., Hirata A., Kawaji T., Fukushima M., Tanihara H. (2007). Autologous plasmin-assisted vitrectomy for stage 5 retinopathy of prematurity: A preliminary trial. Am. J. Ophthalmol..

[248] Hirata A., Takano A., Inomata Y., Yonemura N., Sagara N., Tanihara H. (2007). Plasmin-assisted vitrectomy for management of proliferative membrane in proliferative diabetic retinopathy: A pilot study. Retina.

[249] Wu W.C., Drenser K.A., Capone A., Williams G.A., Trese M.T. (2007). Plasmin enzyme-assisted vitreoretinal surgery in congenital X-linked retinoschisis: Surgical techniques based on a new classification system. Retina.

[250] Esser P., Heimann K., Bartz-Schmidt K.U., Walter P., Krott R., Weller M. (1997). Plasminogen in proliferative vitreoretinal disorders. Br. J. Ophthalmol..

[251] Unal M., Peyman G.A. (2000). The efficacy of plasminogen-urokinase combination in inducing posterior vitreous detachment. Retina.

[252] Hesse L., Nebeling B., Schroeder B., Heller G., Kroll P. (2000). Induction of posterior vitreous detachment in rabbits by intravitreal injection of tissue plasminogen activator following cryopexy. Exp. Eye Res..

[253] Gandorfer A., Rohleder M., Sethi C., Eckle D., Welge-Lüssen U., Kampik A., Luthert P., Charteris D. (2004). Posterior Vitreous Detachment Induced by Microplasmin. Investig. Ophthalmol. Vis. Sci..

[254] Neffendorf J.E., Kirthi V., Pringle E., Jackson T.L. (2017). Ocriplasmin for symptomatic vitreomacular adhesion. Cochrane Database Syst. Rev..

[255] Morescalchi F., Gambicorti E., Duse S., Costagliola C., Semeraro F. (2016). From the analysis of pharmacologic vitreolysis to the comprehension of ocriplasmin safety. Expert Opin. Drug Saf..

[256] Takano A., Hirata A., Ogasawara K., Sagara N., Inomata Y., Kawaji T., Tanihara H. (2006). Posterior vitreous detachment induced by Nattokinase (Subtilisin NAT): A novel enzyme for pharmacologic vitreolysis. Investig. Ophthalmol. Vis. Sci..

[257] Santra M., Sharma M., Katoch D., Jain S., Saikia U.N., Dogra M.R., Luthra-Guptasarma M. (2020). Induction of posterior vitreous detachment (PVD) by non-enzymatic reagents targeting vitreous collagen liquefaction as well as vitreoretinal adhesion. Sci. Rep..

[258] Santra M., Sharma M., Katoch D., Jain S., Saikia U.N., Dogra M.R., Luthra-Guptasarma M. (2021). Enzymatic vitreolysis using reengineered Vibrio mimicus-derived collagenase. Mol. Vis..

