# Supplementary material for: Untangling the Extracellular Matrix of Idiopathic Epiretinal Membrane: A Path Winding among Structure, Interactomics and Translational Medicine
Source: Cells. 2022 Aug 15;11(16):2531. doi: 10.3390/cells11162531 (PMC9406781; doi:10.3390/cells11162531)
Supplement: Supplementary file 1 [file cells-11-02531-s001.zip › cells-1831816-supplementary.pdf]

Supplementary Table and Figures

Table S1: In order to facilitate net understanding, symbols used to represent the 141 experimental selected proteins in the generated nets are listed (column A) near the MetaCore protein names (column B). We also report the corresponding UniProtKB recommended protein names (column C) and identifiers (column D) of the corresponding UniProtKB entries. Column E provides the main compartmental localization of the listed proteins. Edge green-numbers (column F) indicate, for each row-protein, how many interactions are established by our list-proteins with other DIN hubs. Edge red-numbers (column G) specify, for each row-protein, the number of interactions selected iERM-proteins establish within the SPN.

Net symbol	MetaCore Name	UniProtKB Name	UniProtKB ID	Main Localizations	Edges	
	90K	galectin 3 binding protein	LG3BP_HUMAN	membrane	1	16
	A1M	protein AMBP (clived in: Alpha-1-microglobulin, Inter-	AMBP_HUMAN	membrane	0	14
	A2M	alpha-2-macroglobulin	A2MG_HUMAN	extracellular region	5	34
	AGP1 (ORM1)	alpha-1-acid glycoprotein 1 (orosomucoid 1)	A1AG1_HUMAN	extracellular region	0	11
	Agrin	agrin	AGRIN_HUMAN	membrane	1	12
	Alpha 1-antitrypsin	alpha-1-antitrypsin (serpin A1)	A1AT_HUMAN	extracellular region	0	47
	Annexin I	annexin A1	ANXA1_HUMAN	cytoplasm	4	26
	Annexin II	annexin A2	ANXA2_HUMAN	membrane	4	43
	Annexin V	annexin A5	ANXA5_HUMAN	cytoplasm	0	8
	Annexin VI	annexin A6	ANXA6_HUMAN	cytoplasm	3	20
	Annexin VII	annexin A7	ANXA7_HUMAN	cytoplasm	1	11
	Annexin XI	annexin A11	ANX11_HUMAN	cytoplasm	1	15
	Antithrombin III	antithrombin-III	ANT3_HUMAN	extracellular region	0	14
	APCS	serum amyloid P-component	SAMP_HUMAN	extracellular region	0	5
	APOA1	apolipoprotein A1	APOA1_HUMAN	extracellular region	1	30
	APOE	apolipoprotein E	APOE_HUMAN	extracellular region	1	28
	ARG1	arginase-1	ARGI1_HUMAN	cytoplasm	0	23
	AZGP1	zinc-alpha-2-glycoprotein	ZA2G_HUMAN	extracellular region	1	8
	BCAM	basal cell adhesion molecule	BCAM_HUMAN	membrane	1	7
	BETA-IG-H3	transforming growth factor-beta-induced protein ig-h3	BGH3_HUMAN	extracellular region	2	21
	Biglycan	biglycan	PGS1_HUMAN	cytoplasm	2	15
	C1QTNF5	complement C1q tumor necrosis factor-related protein	C1QT5_HUMAN	cytoplasm	0	5
	Calgranulin A	protein S100-A8 (calgranulin-A)	S10A8_HUMAN	cytoplasm	3	19
	Calgranulin B	protein S100-A9 (calgranulin-B)	S10A9_HUMAN	extracellular region	6	29
	Carboxypeptidase A6	carboxypeptidase A6	CBPA6_HUMAN	membrane	0	8
	Cathepsin B	cathepsin B	CATD_HUMAN	cytoplasm	14	51
	Cathepsin D	cathepsin D	CATB_HUMAN	cytoplasm	8	41
	Cathepsin H	pro-cathepsin H	CATH_HUMAN	cytoplasm	3	9
	Cathepsin V	cathepsin L2	CATL2_HUMAN	cytoplasm	2	19
	CD147	basigin	BASI_HUMAN	cytoplasm	6	8
	CD44	CD44 antigen	CD44_HUMAN	membrane	7	94

CHADL	chondroadherin-like protein	CHADL_HUMAN	cytoplasm	0	0
Clusterin	clusterin	CLUS_HUMAN	extracellular region	2	37
COL18A1	collagen alpha-1(XVIII) chain	COIA1_HUMAN	extracellular region	2	31
COL4A1	collagen alpha-1(IV) chain	CO4A1_HUMAN	extracellular region	1	13
COL4A2	collagen alpha-2(IV) chain	CO4A2_HUMAN	extracellular region	0	15
COL4A3	collagen alpha-3(IV) chain	CO4A3_HUMAN	extracellular region	1	7
COL4A4	collagen alpha-4(IV) chain	CO4A4_HUMAN	extracellular region	0	1
COL4A5	Collagen alpha-5(IV) chain	CO4A5_HUMAN	extracellular region	0	1
COL9A2	collagen alpha-2(IX) chain	CO9A2_HUMAN	extracellular region	0	5
Collagen II	collagen alpha-1(II) chain	CO2A1_HUMAN	extracellular region	2	35
Collagen XII	collagen alpha-1(XII) chain	COCA1_HUMAN	extracellular region	0	11
Collagen XIV	collagen alpha-1(XIV) chain	COEA1_HUMAN	extracellular region	1	7
CPA4	carboxypeptidase A4	CBPA4_HUMAN	membrane	0	2
Cystatin A	cystatin-A	CYTA_HUMAN	cytoplasm	4	11
Cystatin B	cystatin-B	CYTB_HUMAN	cytoplasm	1	15
Cystatin C	cystatin-C	CYTC_HUMAN	cytoplasm	3	20
Cystatin SA	cystatin-SA	CYTT_HUMAN	cytoplasm	0	2
Cystatin SN	cystatin-SN	CYTN_HUMAN	cytoplasm	0	2
DMBT1	deleted in malignant brain tumors 1 protein	DMBT1_HUMAN	membrane	0	4
Dystroglycan	dystroglycan 1	DAG1_HUMAN	membrane	0	14
ECM1	extracellular matrix protein 1	ECM1_HUMAN	extracellular region	0	15
EGFR	epidermal growth factor receptor	EGFR_HUMAN	membrane	12	137
EMILIN-1	EMILIN-1	EMIL1_HUMAN	extracellular region	0	12
Endoplasmin	endoplasmin	ENPL_HUMAN	cytoplasm	2	34
EPDR1	mammalian ependymin-related protein 1	EPDR1_HUMAN	extracellular region	0	8
ERp72	protein disulfide-isomerase A4	PDIA4_HUMAN	cytoplasm	0	6
F-spondin	Spondin-1 (Vascular smooth muscle cell growth-promo	SPON1_HUMAN	cytoplasm	0	9
Fascin	fascin	FSCN1_HUMAN	membrane	1	22
Fibrillin 1	fibrillin-1	FBN1_HUMAN	extracellular region	2	20
Fibrinogen alpha	fibrinogen alpha chain	FIBA_HUMAN	extracellular region	0	17
Fibrinogen beta	fibrinogen beta chain	FIBB_HUMAN	extracellular region	0	13
Fibrinogen gamma	fibrinogen gamma chain	FIBG_HUMAN	extracellular region	0	17
Fibronectin	fibronectin	FINC_HUMAN	extracellular region	8	82
Fibulin-3	EGF-containing fibulin-like extracellular matrix protein 1	FBLN3_HUMAN	extracellular region	0	13
FLJ22662	phospholipase B-like 1	PLBL1_HUMAN	cytoplasm	0	8
Galectin-1	galectin-1	LEG1_HUMAN	extracellular region	3	24
Galectin-3	galectin-3	LEG3_HUMAN	cytoplasm	6	42
Gelsolin	gelsolin	GELS_HUMAN	cytoplasm	1	23
Hemopexin	hemopexin	HEMO_HUMAN	extracellular region	1	6
HMGB1	high mobility group protein B1	HMGB1_HUMAN	nucleus	1	61
HSP20	heat shock protein beta-6	HSPB6_HUMAN	cytoplasm	0	5
ICAM1	intercellular adhesion molecule 1	ICAM1_HUMAN	membrane	0	50
IGHV3-13	immunoglobulin heavy variable 3-13	IGHV3-13	cytoplasm	0	0
IL-1F9	interleukin-36 gamma	IL36G_HUMAN	extracellular region	0	7
ILK	integrin-linked protein kinase	ILK_HUMAN	cytoplasm	2	43
ITGA3	integrin alpha-3	ITA3_HUMAN	membrane	3	18
ITGAV	integrin alpha-V	ITAV_HUMAN	membrane	1	29
ITGB1	integrin beta-1	ITB1_HUMAN	membrane	19	78
ITIH5	inter-alpha-trypsin inhibitor heavy chain H5	ITIH5_HUMAN	cytoplasm	0	5
Lacritin	extracellular glycoprotein lacritin	LACRT_HUMAN	extracellular region	0	1
LAMA2	laminin subunit alpha-2	LAMA2_HUMAN	extracellular region	0	4
LAMA4	laminin subunit alpha-4	LAMA4_HUMAN	extracellular region	0	9
LAMA5	laminin subunit alpha-5	LAMA5_HUMAN	extracellular region	1	17
LAMB1	laminin subunit beta-1	LAMB1_HUMAN	extracellular region	0	16
LAMB2	laminin subunit beta-2	LAMB2_HUMAN	extracellular region	0	9
LAMG1	laminin subunit gamma-1	LAMC1_HUMAN	extracellular region	0	18
Lipocalin 1	lipocalin-1	LCN1_HUMAN	extracellular region	0	1
Lipophilin B	secretoglobin family 1D member 2 (Lipophilin-B)	SG1D2_HUMAN	cytoplasm	0	3
LOXL3	lysyl oxidase homolog 3	LOXL3_HUMAN	cytoplasm	0	4
LRP1	prolow-density lipoprotein receptor-related protein 1 (clived in: LRP-85, LRP-515, LRPICD)	LRP1_HUMAN	membrane	9	57
M6B	neuronal membrane glycoprotein M6-b	GPM6B_HUMAN	cytoplasm	0	5
Maspin	serpin B5 (maspin)	SPB5_HUMAN	cytoplasm	2	17
MFGE8	lactadherin (clived in: lactadherin short form, medin	MFGM_HUMAN	extracellular region	0	12
MTS1 (S100A4)	protein S100-A4	S10A4_HUMAN	cytoplasm	2	23
NCAM1	neuronal membrane glycoprotein M6-b	NCAM1_HUMAN	membrane	0	25

	Nidogen	nidogen-1 (entactin)	NID1_HUMAN	membrane	1	19
	Nidogen-2	nidogen-2	NID2_HUMAN	membrane	0	6
	Norrin	norrin	NDP_HUMAN	extracellular region	0	1
	OPTC	opticin	OPT_HUMAN	extracellular region	0	8
	PAI2	plasminogen activator inhibitor 2	PAI2_HUMAN	cytoplasm	0	20
	PAPLN	papilin	PPN_HUMAN	extracellular region	0	1
	PEDF (serpinF1)	pigment epithelium-derived factor	PEDF_HUMAN	extracellular region	1	16
	Perlecan	basement membrane-specific heparan sulfate proteogl	PGBM_HUMAN	extracellular region	0	19
	PKM2	pyruvate kinase PKM (Pyruvate kinase muscle isozyme	KPYM_HUMAN	cytoplasm	3	56
	Plakoglobin	junction plakoglobin	PLAK_HUMAN	cytoplasm	1	25
	Plastin	plastin-2	PLSL_HUMAN	cytoplasm	0	12
	PODN	podocan	PODN_HUMAN	cytoplasm	0	3
	Prolargin	prolargin	PRELP_HUMAN	extracellular region	0	8
	Prosaposin	prosaposin (clived in: saposin A, saposin B, saposin B-	SAP_HUMAN	cytoplasm	2	13
	PRSS11 (HtrA1)	serine protease HTRA1	HTRA1_HUMAN	extracellular region	8	29
	Rac1	Ras-related C3 botulinum toxin substrate 1	RAC1_HUMAN	cytoplasm	4	80
	RNase 7	ribonuclease 7	RNAS7_HUMAN	extracellular region	0	2
	RPESP	somatomedin-B and thrombospondin type-1 domain-co	SBSPO_HUMAN	cytoplasm	0	3
	RS1	retinoschisin	XLRS1_HUMAN	extracellular region	0	4
	S100A10	protein S100-A10	S10AA_HUMAN	membrane	3	21
	S100A13	protein S100-A13	S10AD_HUMAN	cytoplasm	0	4
	S100A7	protein S100-A7 (psoriasin)	S10A7_HUMAN	cytoplasm	1	11
	SCCA-2	serpin B4	SPB4_HUMAN	cytoplasm	0	8
	Serpin B12	serpin B12	SPB12_HUMAN	cytoplasm	0	5
	SERPINA3 (ACT)	alpha-1-antichymotrypsin (Serpin A3)	AACT_HUMAN	cytoplasm	0	26
	SERPINB1	leukocyte elastase inhibitor (Serpin B1)	ILEU_HUMAN	cytoplasm	0	17
	SERPINB6	serpin B6	SPB6_HUMAN	cytoplasm	0	11
	SERPINB8	serpin B8	SPB8_HUMAN	cytoplasm	0	4
	SFRP3 (FRZB1)	secreted frizzled-related protein 3	SFRP3_HUMAN	extracellular region	0	2
	SOD3 (EC-SOD)	extracellular superoxide dismutase [Cu-Zn]	SODE_HUMAN	cytoplasm	1	9
	SPARCL1	SPARC-like protein 1	SPRL1_HUMAN	cytoplasm	0	7
	SRPX	Sushi repeat-containing protein SRPX	SRPX_HUMAN	cytoplasm	0	1
	TARSH	target of Nesh-SH3	TARSH_HUMAN	cytoplasm	0	7
	Tenascin-C	tenascin (cytotactin)	TENA_HUMAN	extracellular region	2	30
	TGM2	protein-glutamine gamma-glutamyltransferase 2	TGM2_HUMAN	cytoplasm	12	69
	Thy-1	thy-1 membrane glycoprotein	THY1_HUMAN	membrane	0	16
	TINAGL1	tubulointerstitial nephritis antigen-like	TINAL_HUMAN	extracellular region	1	7
	Tissue factor	tissue factor (thromboplastin, coagulation factor III)	TF_HUMAN	membrane	2	36
	Trypsin 3	trypsin-3	TRY3_HUMAN	extracellular region	2	9
	Utrophin	utrophin	UTRN_HUMAN	membrane	0	11
	VEGF-A	vascular endothelial growth factor A	VEGFA_HUMAN	extracellular region	5	86
	VEGFR-1	vascular endothelial growth factor receptor 1	VGFR1_HUMAN	membrane	5	43
	Vinculin	vinculin (metavinculin)	VINC_HUMAN	cytoplasm	0	27
	Vitronectin	vitronectin (clived into: vitronectin V65 subunit, vitror	VTNC_HUMAN	extracellular region	2	28
	VWA1	von Willebrand factor A domain-containing protein 1	VWA1_HUMAN	cytoplasm	0	2

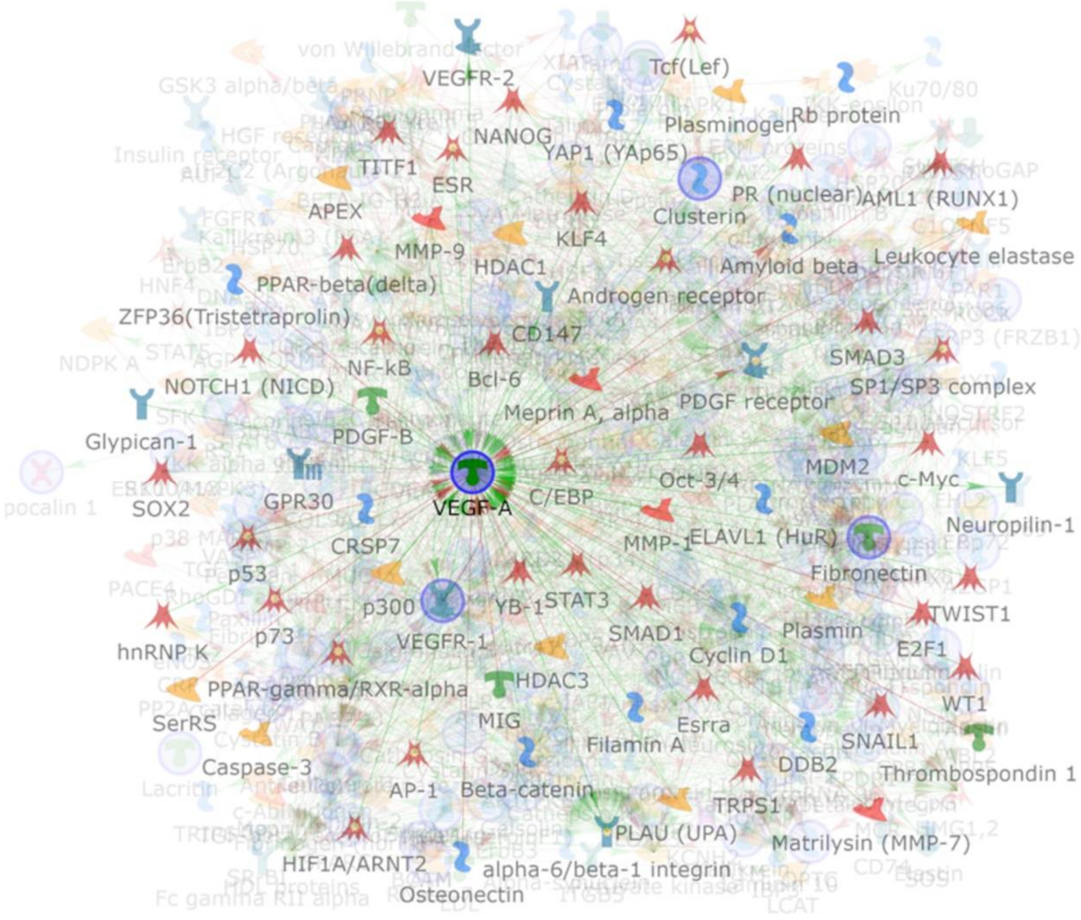

Figure S1: Shortest path net, trace mode visualization centred on VEGF-A. Only proteins that directly interact with VEGF-A (in bold) are visible. The rest of the network appears blurred in the background.

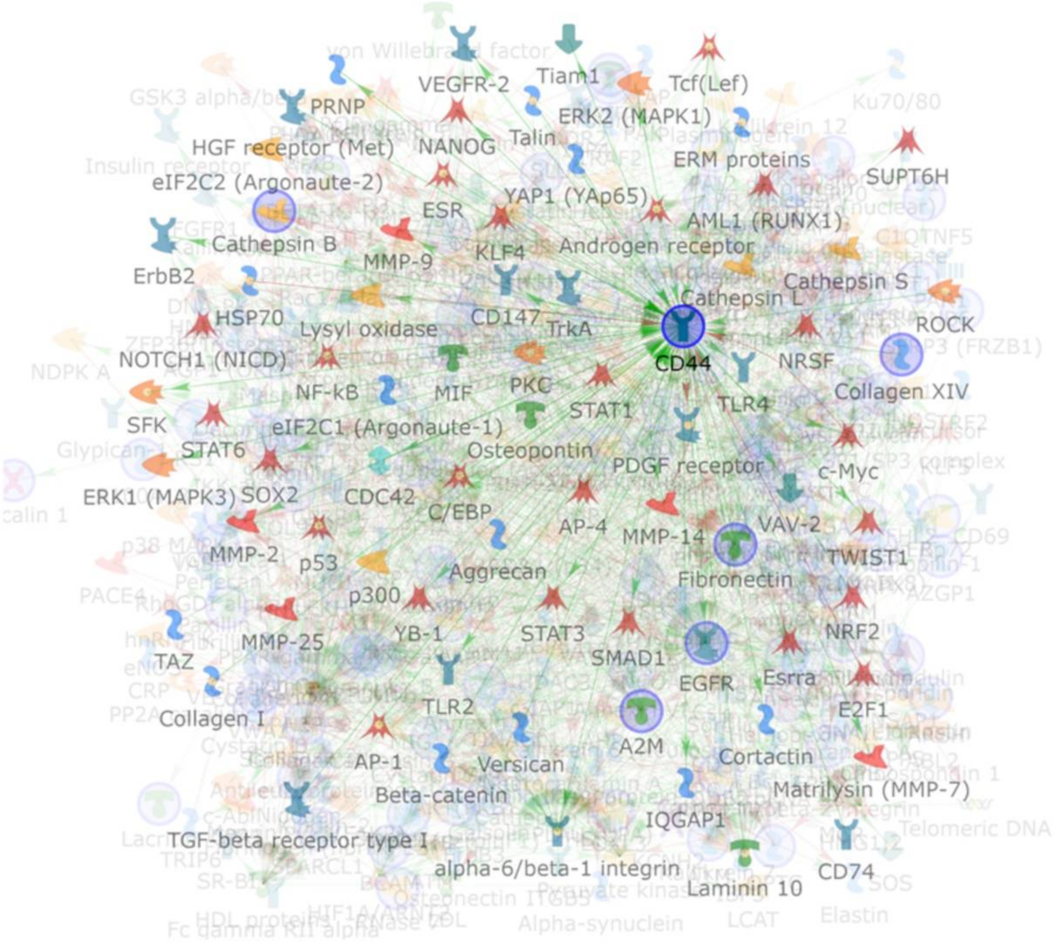

Figure S2. Shortest path net, trace mode visualization centred on CD44. Only proteins that directly interact with CD44 (in bold) are visible. The rest of the network appears blurred in the background.
